# Hydrogen-bonded organic framework for regulating pathogenic autoantigen and oxidative stress in psoriasis treatment

**DOI:** 10.1093/nsr/nwaf132

**Published:** 2025-04-03

**Authors:** Yuhang Li, Hui Yuan, Yi-Lun Cheng, Qiu-Xia Li, Rong Cao, Jin-Lin Li, Qi Yin, Can-Zhong Lu, Tian-Fu Liu

**Affiliations:** State Key Laboratory of Structural Chemistry, Fujian Institute of Research on the Structure of Matter, Chinese Academy of Sciences, China; Xiamen Key Laboratory of Rare Earth Photoelectric Functional Materials, Xiamen Institute of Rare Earth Materials, Chinese Academy of Sciences, China; State Key Laboratory of Structural Chemistry, Fujian Institute of Research on the Structure of Matter, Chinese Academy of Sciences, China; Xiamen Key Laboratory of Rare Earth Photoelectric Functional Materials, Xiamen Institute of Rare Earth Materials, Chinese Academy of Sciences, China; State Key Laboratory of Structural Chemistry, Fujian Institute of Research on the Structure of Matter, Chinese Academy of Sciences, China; University of Chinese Academy of Sciences, China; State Key Laboratory of Structural Chemistry, Fujian Institute of Research on the Structure of Matter, Chinese Academy of Sciences, China; University of Chinese Academy of Sciences, China; State Key Laboratory of Structural Chemistry, Fujian Institute of Research on the Structure of Matter, Chinese Academy of Sciences, China; University of Chinese Academy of Sciences, China; State Key Laboratory of Structural Chemistry, Fujian Institute of Research on the Structure of Matter, Chinese Academy of Sciences, China; College of Chemistry and Materials Science, Fujian Normal University, China; State Key Laboratory of Structural Chemistry, Fujian Institute of Research on the Structure of Matter, Chinese Academy of Sciences, China; University of Chinese Academy of Sciences, China; State Key Laboratory of Structural Chemistry, Fujian Institute of Research on the Structure of Matter, Chinese Academy of Sciences, China; Xiamen Key Laboratory of Rare Earth Photoelectric Functional Materials, Xiamen Institute of Rare Earth Materials, Chinese Academy of Sciences, China; State Key Laboratory of Structural Chemistry, Fujian Institute of Research on the Structure of Matter, Chinese Academy of Sciences, China; University of Chinese Academy of Sciences, China

## Abstract

This study develops a cobalt porphyrin-based hydrogen-bonded organic framework (Co-HOF) to scavenge reactive oxygen species (ROS) and target the pathogenic peptide LL-37, thereby disrupting psoriasis progression.

Psoriasis is an incurable autoimmune disorder that affects ∼2% of the global population [1]. Current therapies primarily target dysregulated immune cytokines and are effective but increase the risk of infections and exacerbate heart failure. Therefore, exploration of strategies that block psoriasis initiation and inflammation amplification while avoiding adverse effects is essential. The formation of psoriatic plaques is closely associated with the antimicrobial peptide LL-37, which exhibits a positively charged surface and is highly expressed in lesions but sparse in healthy skin [2]. LL-37 complexes with self-nucleotides activate dendritic cells (DCs) and keratinocytes, leading to a self-perpetuating inflammatory loop [3,4]. Despite the important role of LL-37 in the initiation of psoriasis and the amplification of inflammation, to the best of our knowledge, the direct elimination of pathologically generated LL-37 has not been explored as a therapeutic approach for psoriasis.

Hydrogen-bonded organic frameworks (HOFs) are a type of porous materials that are self-assembled based on hydrogen bonds and feature good biocompatibility and high crystallinity [5–8]. Moreover, the modular structure allows their physicochemical properties to be tuned and multi-therapeutic approaches to be combined through rational design. In this work, a cobalt porphyrin-based HOF, named Co-HOF, was successfully synthesized. The negatively charged surface and porous structure enable effective trapping and neutralization of LL-37. Moreover, the redox-active building blocks impart the HOF with reactive oxygen species (ROS)-scavenging capacity. Experimental results show that Co-HOF not only neutralizes LL-37, preventing the LL-37-induced activation of DCs and keratinocytes, but also effectively scavenges overgenerated ROS. These features help to resist the inflammatory response in keratinocytes and macrophages. As a result, Co-HOF was able to ameliorate Imiquimod (IMQ)-induced psoriasis-like inflammation in mice without causing obvious side effects.

Co-HOF was prepared through self-assembling [5,10,15,20-Tetrakis(4-carboxyphenyl) porphyrin]-Co(II) (TCPP-Co) by using a solvothermal reaction [8] (Fig. 1A and Fig. S1). Abundant carboxylic acids on the particle surface underwent deprotonation and this led to a negatively charged surface with a zeta potential of –38.06 mV. Unlike many natural peptides that exhibit negatively charged surfaces, LL-37 has a positive surface charge with a zeta potential of +1.57 mV. As shown in Fig. 1B, the addition of LL-37 into the Co-HOF suspension caused a dramatic change in the zeta potential from –20.17 to +24.59 mV, indicating the existence of an interaction between the two components.

**Figure 1.**
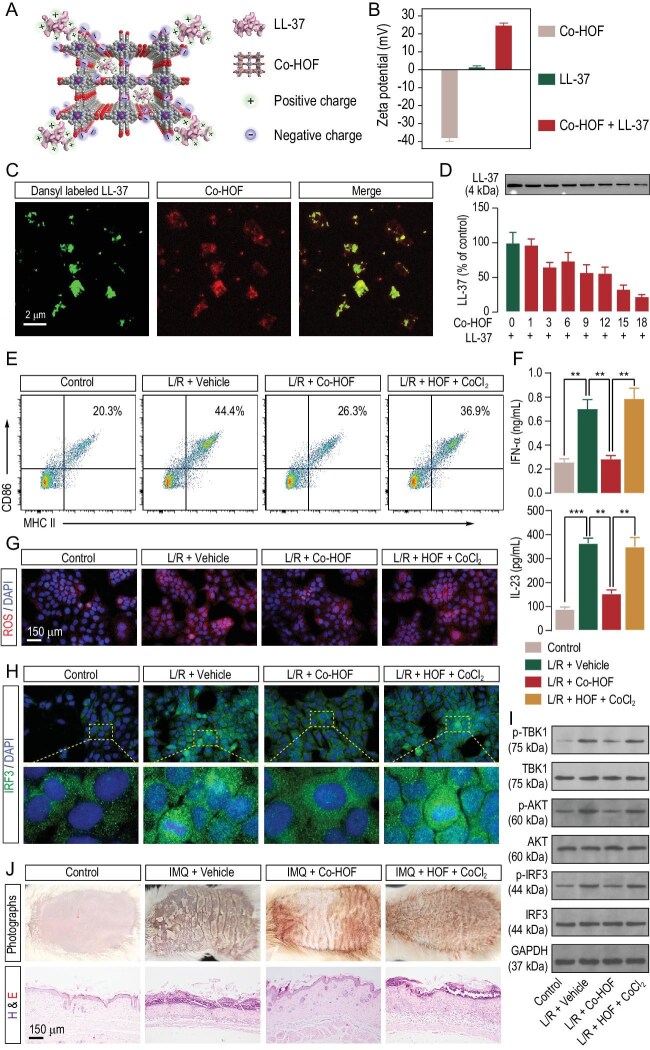
(A) Schematic illustrating the affinity of LL-37 toward Co-HOF driven by electrostatic interaction. (B) The zeta-potential profiles of Co-HOF, LL-37 and their mixture in aqueous solutions. (C) Confocal fluorescence microscopy analysis of Co-HOF in Dansyl-labeled LL-37 aqueous solution; the panels from left to right show the fluorescence images of LL-37, Co-HOF and their emerged image. (D) Western-blotting analyses of the content of free LL-37 in the solution (original: 35 μg/mL) after being treated with varying concentrations of Co-HOF. Data are presented as mean ± standard error of the mean (*n* = 3) throughout the text. (E) Representative flow cytometry of the CD86^+^/MHC-II^+^ cells in bone marrow- derived dendritic cells (BMDCs). (F) The concentrations of interferon (IFN)-α and IL-23 in the BMDCs medium were examined by using enzyme-linked immunosorbnent assay. *P < 0.05; **P < 0.01; ***P < 0.001; these notations apply throughout the text. (G) Representative confocal images of ROS in HaCaT cells. (H) Representative confocal images of the nuclear translocated IRF3 in HaCaT cells. (I) Phosphorylation of TBK1, AKT and IRF3, and expression of total TBK1, AKT and IRF3 in HaCaT cells were assessed by Western-blotting. Glyceraldehyde-3-phosphate dehydrogenase (GAPDH) was used as an internal loading control. (J) Representative skin surface morphology and hematoxylin and eosin staining of the skin samples from the backs of mice.

To gain direct evidence on the attraction of LL-37 in the porous Co-HOF structure, the LL-37 was labeled with a green fluorescent tag of dansyl chloride and mixed with the Co-HOF for 2 hours followed by confocal fluorescence microscopy studies. As shown in Fig. 1C, the fluorescence of the dansyl-labeled LL-37 and the Co-HOF were entirely colocalized. The green fluorescence progressively diminished over 3 days, reflecting the gradual decomposition of the LL-37 in the presence of the Co-HOF. Meanwhile, the red fluorescence was maintained and was not observed in the supernatant, suggesting the robustness of Co-HOF in aqueous solutions (Fig. S11). The treatment of Co-HOF with LL-37 resulted in a dramatic decrease in N_2_ uptake, which might have been caused by the diminished crystallinity of the framework (Figs S2 and S20) and the incorporation of the LL-37 into the cavities of the Co-HOF (18 Å × 18 Å), in view of the larger pore size than the volume of LL-37 (Figs S3 and S8). Due to the extremely low concentration of LL-37, there was no experimental evidence on whether the void space was large enough to encapsulate the LL-37. However, the Co-HOF dose-dependently reduced the LL-37 levels in the solution, demonstrating its effective LL-37-scavenging capacity (Fig. 1D). Confocal microscopy revealed that the Co-HOF prevented LL-37 uptake in HaCaT cells, reducing cytosolic LL-37 aggregation and altering its distribution (Fig. S21). These findings confirm that Co-HOF can block the cellular uptake of LL-37.

The porphyrinic building block imparts framework redox activity for ROS scavenging. ROS, especially H_2_O_2_, superoxide radicals and hydroxyl radicals, are highly reactive chemical compounds that can be generated both exogenously and endogenously, serving as the primary source of oxidative stress. The antioxidant capability of Co-HOF was assessed by using different indicators, confirming that Co-HOF can react with ROS within a short time and at low concentrations (Fig. S12). To determine the origin of the antioxidation activity, cyclic voltammetry studies were performed on the Co-HOF. As shown in Fig. S15, the reduction peak at –0.2 V is lower than the redox potentials of all the oxidative species mentioned above, indicating the thermodynamic feasibility of reducing ROS by using Co-HOF (Table S1). Meanwhile, X-ray photoelectron spectroscopy studies on Co-HOF showed that only a small amount of Co^2+^ was transformed into Co^3+^, ruling out a stoichiometric reaction between the Co-HOF and reactive oxygen species (Fig. S16). We also prepared an isostructural porphyritic HOF without Co ions chelating in the center (Fig. S1, Section 2.2, named free-base HOF), which could barely eliminate ROS under similar conditions. These results indicate that ROS elimination is a catalytic reaction that involves proton-coupled electron transfer from the Co-HOF to the oxidant, followed by regeneration to its initial oxidation state in an ambient environment.

DCs can be activated and matured by the LL-37 and ribonucleic acid (RNA) complex [9] and Co-HOF was found to be stable in the psoriasis wound environment (Fig. S19). By scavenging LL-37 and ROS, Co-HOF can reduce the expression of CD86 and MHC-II (Fig. 1E and Fig. S27), suppressing the maturation of DC and the production of pro-inflammatory cytokines in these cells (Fig. 1F and Fig. S28). It has been well established that cytokines that are released by matured DCs can polarize resident M0 macrophages into M1 phenotype macrophages with an excessive production of pro-inflammatory factors and ROS, leading to the secondary expansion of psoriatic inflammation. Co-HOF significantly inhibited the macrophage M1 polarization that was stimulated by conditioned media (CM) from matured DCs, as reflected by the decreased expression of the biomarkers CD68 and CD86 (Fig. S30) and lower levels of pro-inflammatory cytokines and ROS (Figs S31 and S32). Considering the essential role of the NF-κB and STAT3-RORγT pathways in psoriasis inflammation, we hypothesized that Co-HOF may ameliorate the inflammatory responses of macrophages through regulating these pathways [4,9]. As expected, CM stimulation not only facilitated the phosphorylation of p65 and IκBα (a sign of NF-κB pathway activation), but also promoted the expression of p-STAT3 and RORγT, serving as a strong driving force for the macrophage polarization (Fig. S33). Co-HOF inhibited the activation of these pathways and therefore reduced CM-induced inflammatory responses in the macrophages. Notably, the physical mixture of free-base HOF and CoCl_2_ showed no such effects (Fig. S33), supporting the vital role of the redox-active backbone and the synergistic effect.

Keratinocytes play a pivotal role in the onset and maintenance of DC maturation in psoriasis [10]. They can be activated by an LL-37 and RNA complex, resulting in the hyperproliferation of keratinocytes and overexpression of ROS and various inflammatory cytokines, which could promote the maturation of DC and the secondary expansion of psoriasis inflammation. Treatment with Co-HOF significantly reduced these inflammatory responses (Fig. 1G and Figs S22–S24). Furthermore, keratinocyte activation in psoriasis is mediated by the TBK1–AKT–IRF3 pathway [10]. We hypothesized that Co-HOF inactivates keratinocytes by inhibiting TBK1–AKT–IRF3 signaling following the scavenging of LL-37 and ROS. As expected, Co-HOF attenuated LL-37 and RNA-induced phosphorylation of TBK1, AKT, and IRF3 in HaCaT cells (Fig. 1I and Fig. S35). Additionally, Co-HOF reduced IRF3 nuclear translocation, leading to decreased production of IFNs and other inflammatory cytokines (Fig. 1H and Figs S24 and S25). In contrast, a mixture of free-base HOF and CoCl_2_ did not affect TBK1–AKT–IRF3 signaling. Our data thus far demonstrate that Co-HOF not only prevents the initiated keratinocytes activation and DC maturation, but also attenuates subsequent macrophage polarization and immune cytokine production. Therefore, we speculated that the inhibition of immune-cell activities by Co-HOF ameliorates subsequent psoriasis development.

To test this hypothesis, we then assessed the therapeutic effect of Co-HOF in IMQ-induced psoriasis-like inflammation in mice (Fig. S37). Topical application of Co-HOF alleviated IMQ-induced psoriasis-like features, including erythema, scaling, epidermal thickening and immune-cell infiltration (Fig. 1J and Fig. S38). The hyperproliferation of keratinocytes, infiltration of immune cells and overproduction of pro-inflammatory factors are typical symptoms of psoriasis inflammation [3]. Co-HOF reduced keratinocyte hyperproliferation (Ki-67^+^ cells), CD11c^+^ macrophage/DC infiltration and TNFα production (Fig. S39). Besides, no significant organ injuries were observed, indicating the good biocompatibility of Co-HOF (Fig. S41). Free-base HOF and CoCl_2_ showed no comparable effects (Figs S38 and S39). Collectively, our findings demonstrate the potential of Co-HOF in ameliorating IMQ-induced psoriasis-like inflammation by scavenging LL-37 and ROS. In addition, RNA sequencing revealed 416 differentially expressed genes in Co-HOF-treated psoriatic skin, with downregulating pro-inflammatory genes (e.g. IL-1β) and upregulating anti-inflammatory Serpina3K (Fig. S42). Kyoto Encyclopedia of Genes and Genomes analysis implicated the role of Co-HOF in suppressing the IL-23/IL-17 axis, TNF-α signaling and cell-proliferation pathways (Figs S43 and S44). These findings underscore the potential of Co-HOF as an oxidative stress and inflammation regulator in psoriasis treatment.

In summary, we report a metalloporphyrin hydrogen-bonded organic framework to scavenge pathogenic drivers of psoriasis. The negatively charged surface and void space endow Co-HOF with a high affinity toward autoantigen LL-37, leading to its neutralization and thereby preventing the activation of DC and keratinocytes as well as the subsequent secretion of immune factors. Meanwhile, the porphyrinic backbones impart material redox activity for eliminating excess generated ROS, thus combating the inflammatory responses in keratinocytes and macrophages. In this way, Co-HOF can attenuate the M1 polarization of macrophages induced by the DC-released immune factors, alleviating psoriasis-like inflammation in mice without significant adverse effects. This study develops a strategy for designing efficient molecule-based porous materials to scavenge pathogenic drivers as anti-psoriasis agents and provides new insights for developing novel treatments for other autoinflammatory diseases.

## Supplementary Material

nwaf132_Supplemental_File

## Data Availability

All data generated or analysed during this study are included in this published article and its Supplementary information files. Crystallographic data for the structures reported in this article have been deposited at the Cambridge Crystallographic Data Centre, under deposition numbers CCDC 2107777 (free-base HOF) and 2107780 (Co-HOF). Copies of the data can be obtained free of charge via https://www.ccdc.cam.ac.uk/structures/.
