# Supplementary material for: Hydrogen-bonded organic framework for regulating pathogenic autoantigen and oxidative stress in psoriasis treatment
Source: Natl Sci Rev. 2025 Apr 3;12(6):nwaf132. doi: 10.1093/nsr/nwaf132 (PMC12080220; doi:10.1093/nsr/nwaf132)
Supplement: nwaf132_Supplemental_File [file nwaf132_supplemental_file.zip › Supplementary data.pdf]

Supporting Information

Hydrogen-Bonded Organic Framework for Regulating Pathogenic Autoantigen and Oxidative Stress in Psoriasis Treatment

Contents

Experimental section......3

1.1 Materials......3

1.2 Characterizations......3

2.1 Syntheses of TCPP and TCPP-Co[1]......4

2.2 Syntheses of powdery samples of free-base HOF and Co-HOF......6

2.3 N_2 absorption isotherms of Co-HOF......6

2.4 The self-assembly processes and structures of free-base HOF and Co-HOF......7

2.5 Scanning electron microscopy (SEM) measurements......8

2.6 Peptide binding assay and confocal fluorescence microscopy analyses......9

2.7 Antioxidant capacity of Co-HOF......11

2.8 H_2O_2 scavenging activity assay.......11

2.9 •OH scavenging activity assay.......12

2.10 $O_2^{•-}$ scavenging activity assay......12

2.11 Vc scavenging activity assay......12

2.11 CV curves measurement......15

2.12 X-ray photoelectron spectroscopy (XPS) measurements......16

2.13 Thermogravimetry (TG) curve......16

2.14 Stability test of Co-HOF......17

2.15 HaCaT keratinocytes culture and stimulation......19

2.16 Bone marrow-derived dendritic cells (BMDCs) culture and stimulation......22

2.17 RAW264.7 macrophage culture and stimulation......24

2.18 Cell viability......27

2.19 ROS assay......27

2.20 EdU assay......27

2.21 Western blotting......28

2.22 Reverse transcription-quantitative PCR (RT-qPCR) 29

2.23 Enzyme linked immunosorbent assay (ELISA) 29

2.24 Flow cytometry assay 29

2.25 Animal 30

2.26 Imiquimod (IMQ)-induced mouse model of psoriasis 30

2.27 Histology 32

2.28 Immunofluorescence 33

2.29 RNA sequencing (RNA-seq) analysis 34

3 Data and statistical analysis 36

Supplemental References 36

Experimental section

1.1 Materials

N, N-dimethylformamide (DMF, AR), *N, N*-dimethylacetamide (DMA, AR), dimethyl sulfoxide (DMSO, AR), propionic acid (AR), triethylamine (AR), methanol (MeOH, AR), ethanol (EtOH, AR), acetone (AR) and cobalt (II) chloride dihydrate (99%), hydrogen peroxide (H_2O_2), 2,2'-Azinobis-(3-ethylbenzthiazoline-6-sulphonate) (ABTS), ferrous sulfate ($FeSO_4$), 2,2-Diphenyl-1-(2,4,6-trinitrophenyl) hydrazyl (DPPH), 5,5-Dimethyl- 1-pyrroline N-oxide (DMPO), 2-Phennyl- 4,4,5,5-tetramethylimidazoline-q-oxyl 3-Oxide (PTIO), β-Nicotinamide Adenine Dinucleotide, Reduced Disodium Salt Hydrate, Phenazine Methosulfate, Dansyl Chloride, 1,2,4-Trichlorobenzene were purchased from Adamas. Unless otherwise specified, all reagents and solvents were purchased from commercial sources and used as received without further purification.

1.2 Characterizations

The powder X-ray diffraction (PXRD) patterns were recorded using a Rikagu Miniflex 600 Benchtop and Rikagu Smartlab equipped with Cu Kα radiation (λ = 1.54056 Å). The N_2 isotherms were measured using an ASAP 2460 from Micromeritics Co. Ltd. Thermogravimetric analysis (TGA) was tested on a Netzsch instrument with samples being heated at a rate of 5 °C/min up to 800 °C under N_2 atmosphere. UV-Visible absorbance spectra were collected at room temperature on a Shimadzu UV-2550 spectrophotometer. Steady-state photoluminescence spectra were measured using FS5 fluorescence spectrophotometer under ambient condition. ^{1}H-NMR spectra were recorded on Bruker AVANCE III 400 MHz spectrometers. The zeta potentials were collected by BI-200SM Analyzer or NanoBrook omni dynamic light scattering (DLS) particle size and zeta potential analyzer (Brookhaven) following the manufacturer's instructions. Electron paramagnetic resonance (EPR) measurements were performed at X-band (~9.8 GHz) using a Bruker Bruker-BioSpin EPR spectrometer at room temperature. The X-ray photoelectron spectroscopy (XPS) data were collected by ESCALAB 250Xi. The Scanning electron microscopy (SEM) images were obtained by the Phenom G2 Field Emission Scanning Electron Microscope and equipped Energy Dispersive Spectroscopy (EDS) system at an acceleration voltage of 15 kV. The Confocal fluorescence microscopy images were obtained by the Nikon C2. The electrochemical test was performed on an electrochemical analyzer (Zahner, Germany). X-ray photoelectron spectroscopy (XPS) measurements were performed on a Thermo Fisher ESCALAB 250Xi spectrometer with Al Kα X-ray source (15 kV, 10 mA) with C 1s peak at 284.6 eV as internal standard.

2.1 Syntheses of TCPP and TCPP-Co[1]

5,10,15,20-Tetrakis(4-methoxycarbonylphenyl) porphyrin (TPPCOOMe)

To refluxed propionic acid (100 mL) in a 500 mL three necked flask, pyrrole (6.0 g, 0.086 mol) and methyl p-formylbenzoate (12 g, 0.086 mol) were added, and the solution was refluxed for 12 h in darkness. After cooling down the reaction mixture to room temperature, crystals were collected by suction-filtration to afford purple crystals (3.6 g, 4.24 mmol, 20.2 % yield). ^{1}H NMR (400 MHz, $CDCl_3$) δ 8.81 (s, 8H), 8.43 (d, 8H), 8.28 (d, 8H), 4.11 (s, 12H), -2.83 (s, 2H).

5,10,15,20-Tetrakis(4-carboxyphenyl) porphyrin (TCPP)

TPPCOOMe (2 g, 2.71 mmol) was stirred in THF (80 mL) and MeOH (80 mL) mixed solvent, to which a solution of KOH (8.42 g, 150.24 mmol) in H_2O (80 mL) was introduced. This mixture was refluxed for 12 h. After cooling down to room temperature, THF and MeOH were removed through evaporation. Additional 200 mL water was added until the solid was fully dissolved, then the homogeneous solution was acidified with 1 M HCl until no further precipitate was detected. The purple solid was collected by filtration, washed with water and dried in vacuum (1.8 g, 2.61 mmol, 96 % yield). ^{1}H NMR (400 MHz, DMSO-d6) δ 13.22 (s, 4H), 8.87 (s, 8H), 8.40 (d, 8H), 8.35 (d, 8H), -2.94 (s, 2H).

[5,10,15,20-Tetrakis(4-carboxyphenyl) porphyrinato]-Co(II) (TCPP-Co)

A solution of TPPCOOMe (0.854 g, 1.0 mmol) and $CoCl_2 \cdot 6H_2O$ (3.1 g, 12.8 mmol) in 100 mL of DMF was refluxed for 8 h. After the mixture was cooled to room temperature, 150 mL of H_2O was added. The resultant precipitate was filtered and washed with 50 mL of H_2O twice. The obtained solid was dissolved in $CHCl_3$, followed by washing three times with water. The organic layer was dried over anhydrous magnesium sulfate and evaporated to afford red powder. The obtained powder (0.75 g) was stirred in THF (25 mL) and MeOH (25 mL) mixed solvent, to which a solution of KOH (2.63 g, 46.95 mmol) in H_2O (25 mL) was introduced. This mixture was refluxed for 12 h. After cooling down to room temperature, THF and MeOH were removed through evaporation. Additional 200 mL water was added to the resulting water phase until the solid was fully dissolved, then the homogeneous solution was acidified with 1M HCl until no further precipitate was detected. The red solid was collected by filtration, washed with water and dried in vacuum (0.61 g, 0.720 mmol, 87.5% yield).

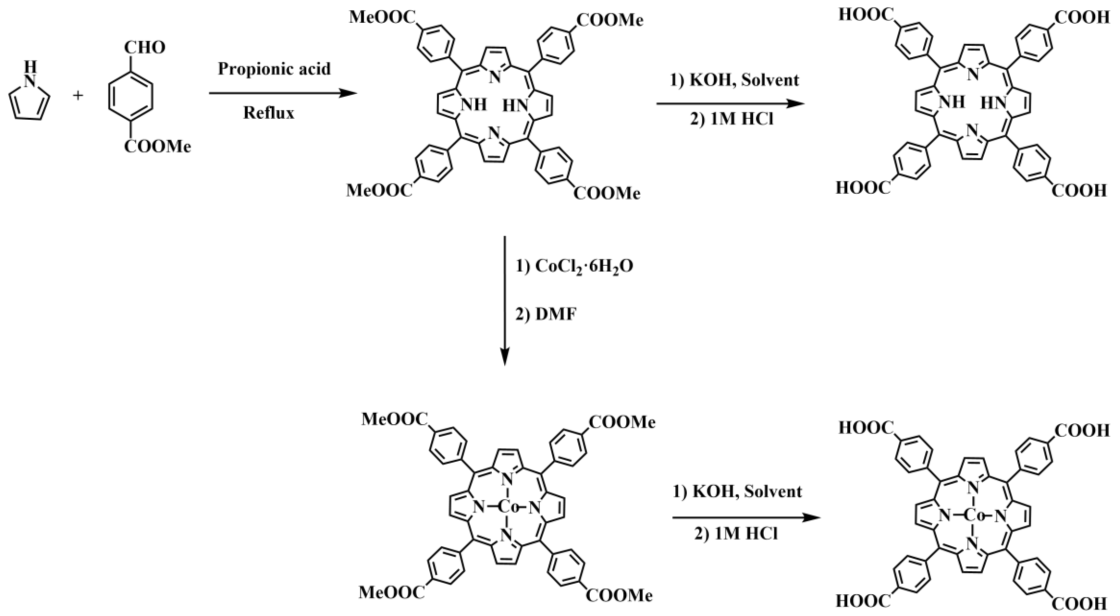

Figure S1. Synthetic routes for TCPP and TCPP-Co.

2.2 Syntheses of powdery samples of free-base HOF and Co-HOF

TCPP (20 mg, 0.029 mmol) or TCPP-Co (30 mg, 0.039 mmol) was dissolved in 9 mL CH_3COOH in a 15 mL capped glass bottle. Then the capped glass bottle was held at 90 °C for 2 days, the powdery samples free-baseHOF (15 mg, 75 % yield) or Co-HOF (22 mg, 73 % yield) were harvested.

2.3 N_2 absorption isotherms of Co-HOF

The as-prepared sample was washed with CH_2Cl_2 3 times. Then the sample was allowed to soak in CH_2Cl_2 for 96 h with the supernatant being replaced by fresh CH_2Cl_2 several times during the process to exchange and remove nonvolatile solvates (TCB, H_2O and DMF). After removal of CH_2Cl_2 by centrifugation, the samples were activated under vacuum at room temperature, and then dried again in the "outgas" function of instruments at 100 °C for 8 hours for Co-HOF prior to gas adsorption. The N_2 isotherm measurements were performed at 77 K and a pressure of 1 bar.

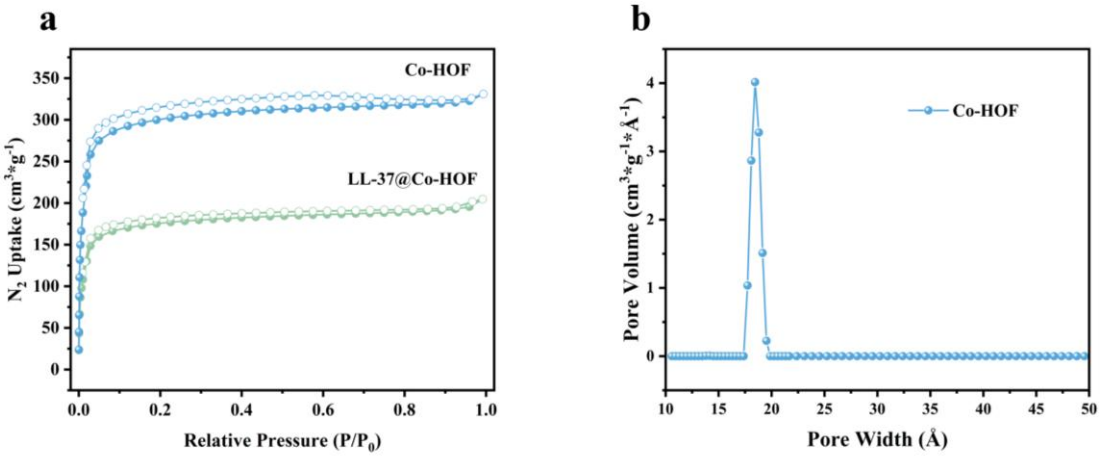

Figure S2. a) N_2 isotherms of Co-HOF and LL-37@Co-HOF. b) Pore size analyses of Co-HOF.

2.4 The self-assembly processes and structures of free-base HOF and Co-HOF

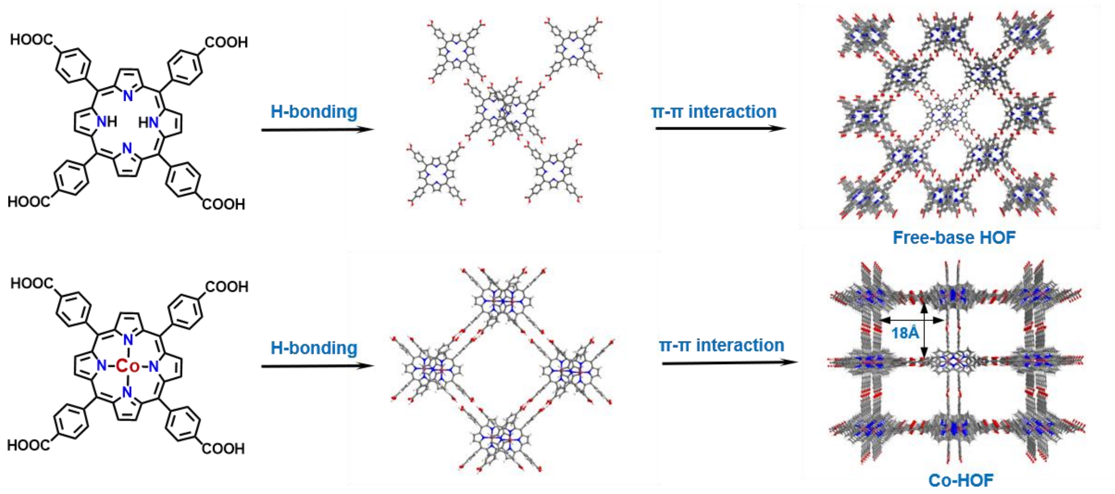

Figure S3. Crystal structure of free-base HOF and Co-HOF.

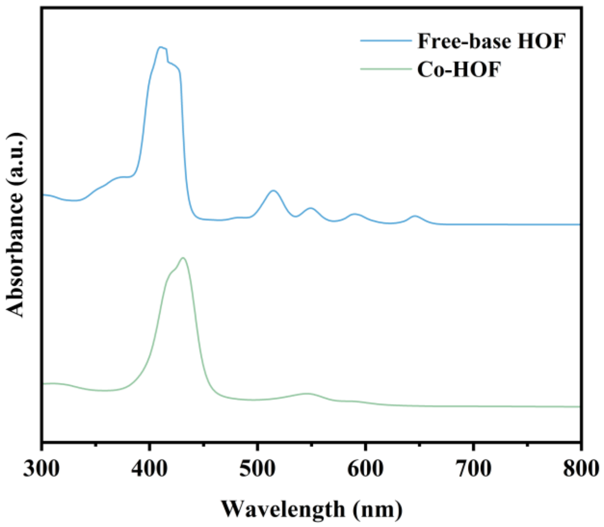

Figure S4. The UV absorption spectrum of Co-HOF and free-base HOF. The coordination of a cobalt(II) ion within porphyrin induces a characteristic modification in the Q-band electronic absorption spectrum, manifesting as a reduction in vibrational fine structure complexity with 2-3 fewer distinct peaks observed in the spectral region centered around 600 nm.[1, 2]

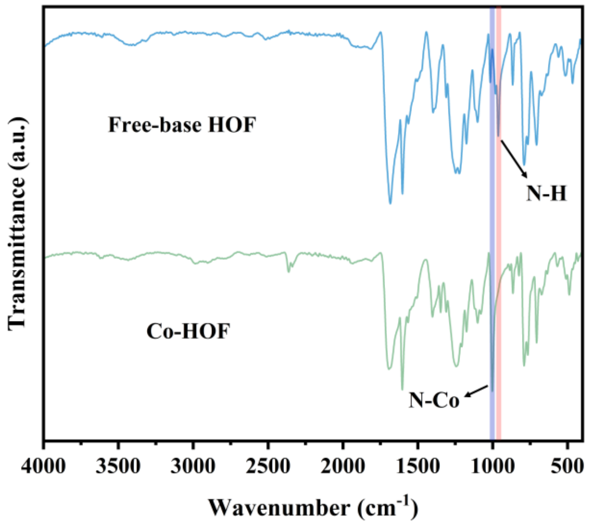

Figure S5. The FTIR spectrum of Co-HOF and free-base HOF. The distinct separation in characteristic infrared stretching frequencies between the ν(Co-H) and ν(N-H) vibrational modes provides conclusive spectroscopic evidence for the central coordination of cobalt within the porphyrin macrocycle, as the observed wavenumber differential directly correlates with the unique electronic environment generated by metalloporphyrin complexation.

2.5 Scanning electron microscopy (SEM) measurements

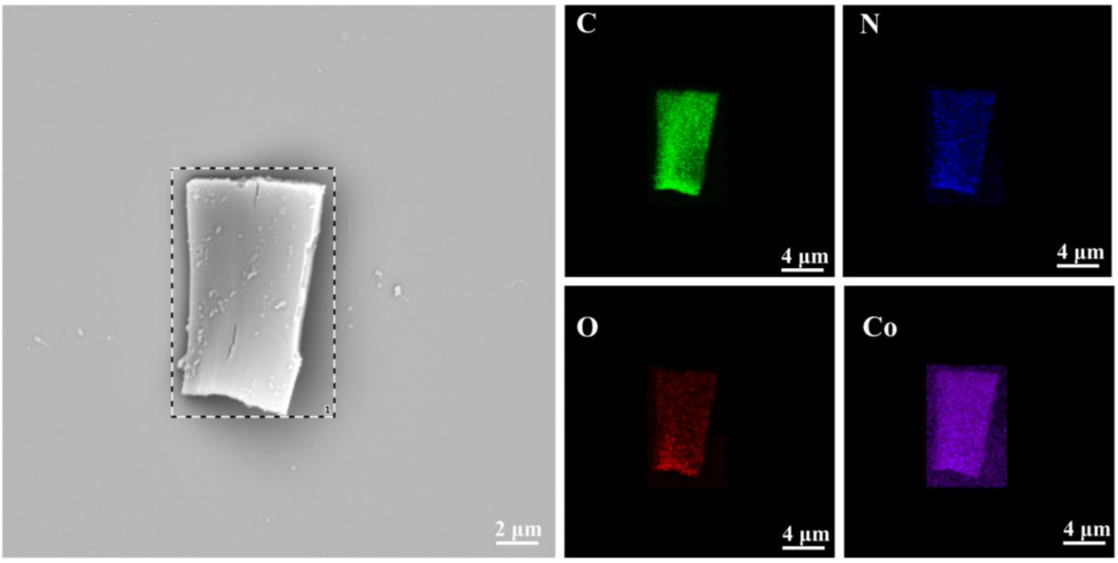

Figure S6. SEM images and the energy-dispersive X-ray spectroscopy (EDS) elemental mapping of Co-HOF images.

2.6 Peptide binding assay and confocal fluorescence microscopy analyses

First, we measured the zeta potentials of free-base HOF (7 μg/mL), Co-HOF (7 μg/mL), LL-37 (35 μg/mL) in aqueous solution, as well as the physical mixture of HOF (7 μg/mL) and LL-37 (35 μg/mL), Co-HOF (7 μg/mL) and LL-37 (35 μg/mL).

Dansyl labeled LL-37 was synthesized by mixing 10 mL LL-37 solution (50 μg/mL in deionized water) with 2 mL dansyl chloride solution (2.5 mg/mL in acetone) in the presence of triethylamine (0.15 mL) and stirring at room temperature for 5 hours. After dialysis and measuring the protein concentration with ELISA kit, the dansyl labeled LL-37 was obtained for next step uses.

Subsequently, free-base HOF, Co-HOF, LL-37 and dansyl chloride labeled LL-37 were tested separately on a fluorescence spectrometer. Two sets of 1 mL Co-HOF aqueous solution (0.5 mg/mL) were respectively combined with 1 mL of dansyl chloride-labeled LL-37. One set of the mixed solution was applied to a glass slide, allowed to dry, and subsequently photographed using a confocal fluorescence microscope. The other set was stood at room temperature and then subjected to confocal fluorescence microscope analysis. The same operation to free-base HOF (0.5 mg free-base HOF suspended in 1 mL water) result in immediate structural decomposition as observed by confocal microscope. (Co-HOF: λ_{ex} = 420 nm, λ_{em} = 660 nm; HOF: λ_{ex} = 465 nm, λ_{em} = 750 nm; dansyl labeled LL-37: λ_{ex} = 376 nm, λ_{em} = 494 nm)

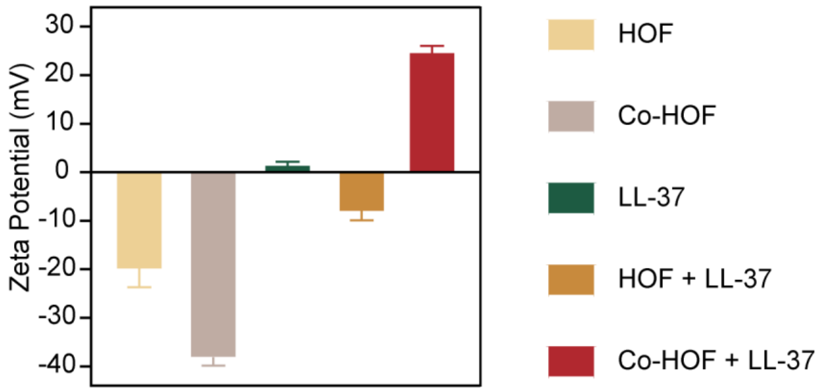

Figure S7. The zeta potential profile of free-base HOF, Co-HOF, LL-37, and their mixture in aqueous solutions.

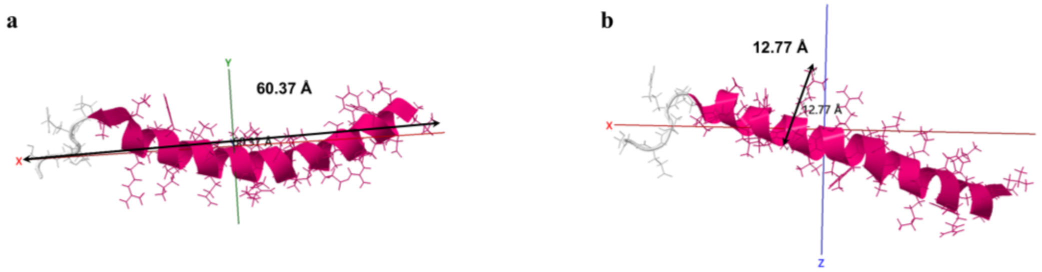

Figure S8. Schematic representation of LL-37 structures from different directions.

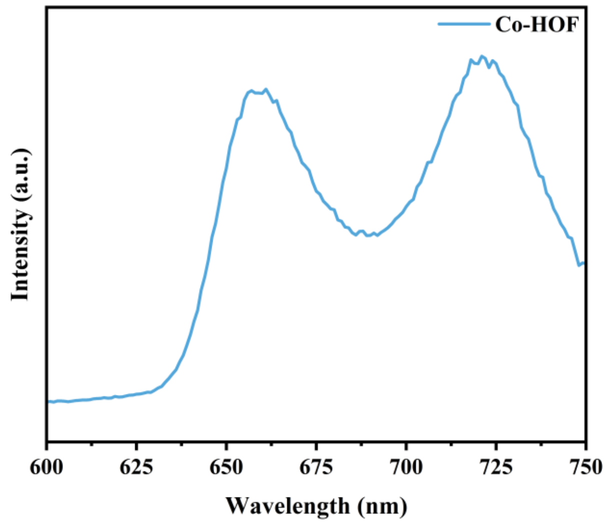

Figure S9. Fluorescence spectra of Co-HOF.

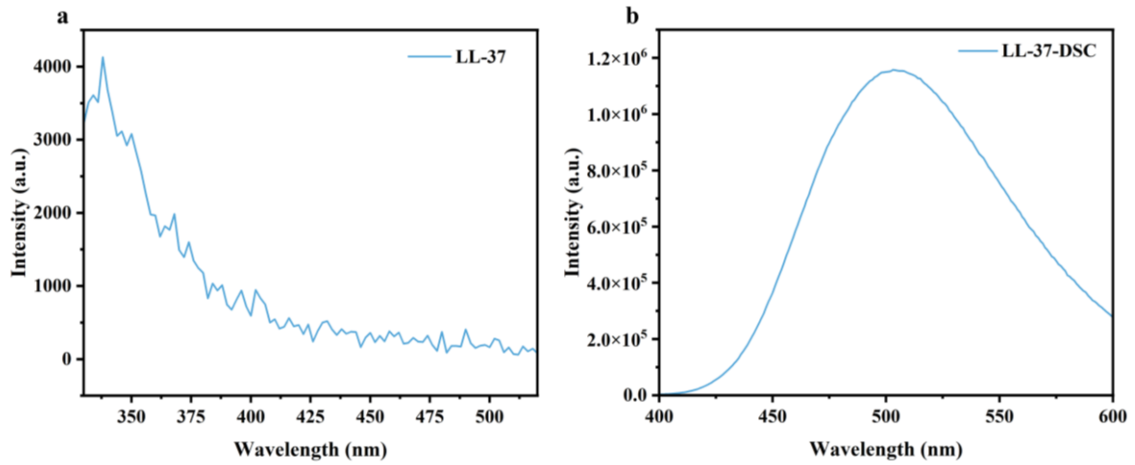

Figure S10. Fluorescence spectra of LL-37 and dansyl chloride labeled LL-37.

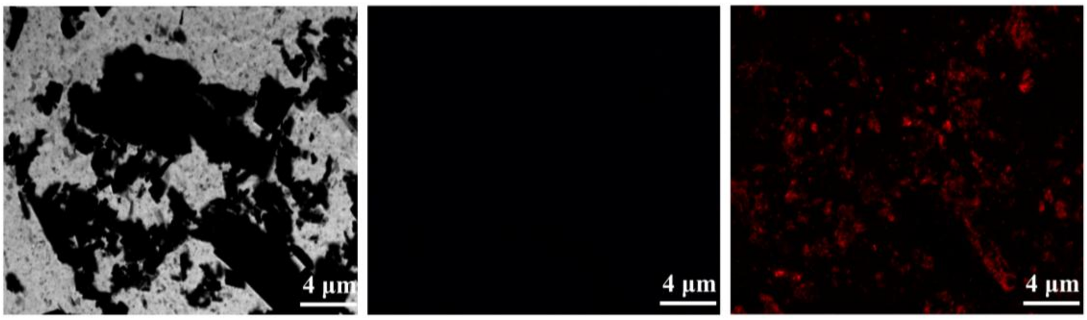

Figure S11. Confocal fluorescence microscopy images of Co-HOF immersing in dansyl chloride-labeled LL-37 solution for 3 days. From left to right the panel shows the fluorescence from brightfield, LL-37, and Co-HOF.

2.7 Antioxidant capacity of Co-HOF

2, 2'-Azino-bis (3-ethylbenzothiazoline-6-sulfonic acid) (ABTS)[[3]], 2,2-diphenyl-1-picrylhydrazyl (DPPH) [[4]] and Phennyl-4,4,5,5-tetramethylimidazoline-q-oxyl 3-Oxide (PTIO)[[5]] were used to measure the total antioxidant capacity of Co-HOF. The $ABTS^{+\bullet}$ solution was prepared according to the literature method in which potassium persulfate was used as the oxidant for ABTS to obtain $ABTS^{+\bullet}$ radicals. The detailed method: ABTS (0.068 mmol) powder was dissolved into 30 mL deionized water, followed by the addition of $K_2S_2O_8$ (0.024 mmol) under stirring. The reaction (pH = 7) was left to proceed in the dark at 25 °C for 12 h. To test the scavenging capacity of Co-HOF toward $ABTS^{+\bullet}$, the antioxidant activity, 1 mL Co-HOF in deionized water with varying concentrations (0.2, 0.4, 0.6, 0.8, 1.0 mg/mL) was added to 1 mL $ABTS^{+\bullet}$ solution. Then the reduction of ABTS was determined by monitoring its absorption at 417 and 734 nm. To test the DPPH• scavenging activity, 0.5 mL of 0.2 mg/mL DPPH• acetonitrile solution was combined with 0.5 mL of deionized water. Then, 1 mL Co-HOF in water suspension with different concentration (10, 20, 30, 40, 50 µg/mL) of were added. Two minutes later, the absorbance at 519 nm were recorded by UV-Vis spectrometer to evaluate the reduction rate of DPPH•. For PTIO•, 1 mL Co-HOF in water suspension with different concentrations (0.1, 0.2, 0.3, 0.4, 0.5 mg/mL) was added to 1 mL of 0.1 mg/mL PTIO• aqueous solution. The absorbance at 557 nm were recorded by UV-Vis spectrometer to measure the remaining PTIO• in solution. The same operations were conducted to determine the anti-oxidation of HOF with the concentration of 0.5 mg/mL in water.

2.8 H_2O_2 scavenging activity assay.

The H_2O_2 scavenging activity was measured by titanium sulfate colorimetric method. To the mixture of $Ti(SO_4)_2$ (1 mmol), H_2SO_4 (4 mL), and Co-HOF (20, 40, 60, 80, 100 µg/mL), free-base HOF (0.5 mg/mL) or TCPP-Co (0.01, 0.02, 0.03, 0.04 mg/mL) in deionized water (16 mL), a solution of H_2O_2 (3 mM) in deionized water (10 mL) was added and stirred for 2 min. The reduction of H_2O_2 was monitored by measuring the absorbance changes of the formed peroxo titanic acid using UV-Vis spectrometer. The

same operations were conducted to determine the H_2O_2 scavenging activity of free-base HOF with concentration of 0.5 mg/mL in water.

2.9 •OH scavenging activity assay.

•OH was generated by a classical Fenton reaction between Fe^{2+} and H_2O_2, and its production and removal process were tested by ESR. To the mixture of DMPO (0.25 μmol), H_2O_2 (0.1 μmol) and $FeSO_4$ (0.1 μmol) in 50 μL deionized water, 20 μL suspension of 50 μg/mL or 100 μg/mL Co-HOF in deionized water were added, respectively. After incubating for 5 min, the ESR spectra of resulting mixture were recorded to determine the DMPO/•OH adducts. The same operations were conducted to determine the •OH scavenging activity of HOF with concentration of 0.5 mg/mL in water.

2.10 $O_2^{\bullet-}$ scavenging activity assay

$O_2^{\bullet-}$ scavenging activity was determined by the nicotinamide adenine dinucleotide (NADH)-phenazine methosulfate (mPMS)-nitroblue tetrazolium (NBT) method. The reaction between NADH and mPMS can produce $O_2^{\bullet-}$, which can oxidize NBT with the characteristic UV absorption peak appearing at 680 nm. To the mixture of NADH (0.019 mmol), mPMS (6.8 μmol), Co-HOF (0.1, 0.2, 0.3, 0.4, 0.5 mg/mL) or free-base HOF (0.5 mg/mL) in deionized water (900 μL), a solution of NBT (5.8 μmol) in deionized water (100 μL) was added. After incubating for 5 min, the UV-Vis spectra of resulting mixture were recorded to measure the remaining oxidative form of NBT at 680 nm. The same operations were conducted to determine the $O_2^{\bullet-}$ scavenging activity of HOF with concentration of 0.5 mg/mL in water.

2.11 Vc scavenging activity assay

Vitamin C (Vc) was used as a positive control to confirm that the experimental setup can detect positive results. To test the scavenging capacity of Vc toward $ABTS^{+\bullet}$, the antioxidant activity, 1 mL Vc in deionized water with varying concentrations (0.05, 0.10, 0.15, 0.20, 0.25, 0.30 mg/mL) was added to 1 mL $ABTS^{+\bullet}$ solution. Then the reduction of ABTS was determined by monitoring its absorption at 417 and 734 nm.

All above ROS scavenging experimental procedures were conducted under controlled temperature conditions at 25°C.

Equation used for calculating the removal of ROS or ROS mimics:

$$\text{Elimination (\%)} = \frac{[(A_0 - A_1)]}{A_0} \times 100\% \quad (1)$$

where, A_0 is the absorbance of initial ROS or ROS mimics, and A_1 is the absorbance of ROS in the presence of HOF or Co-HOF after a certain period.

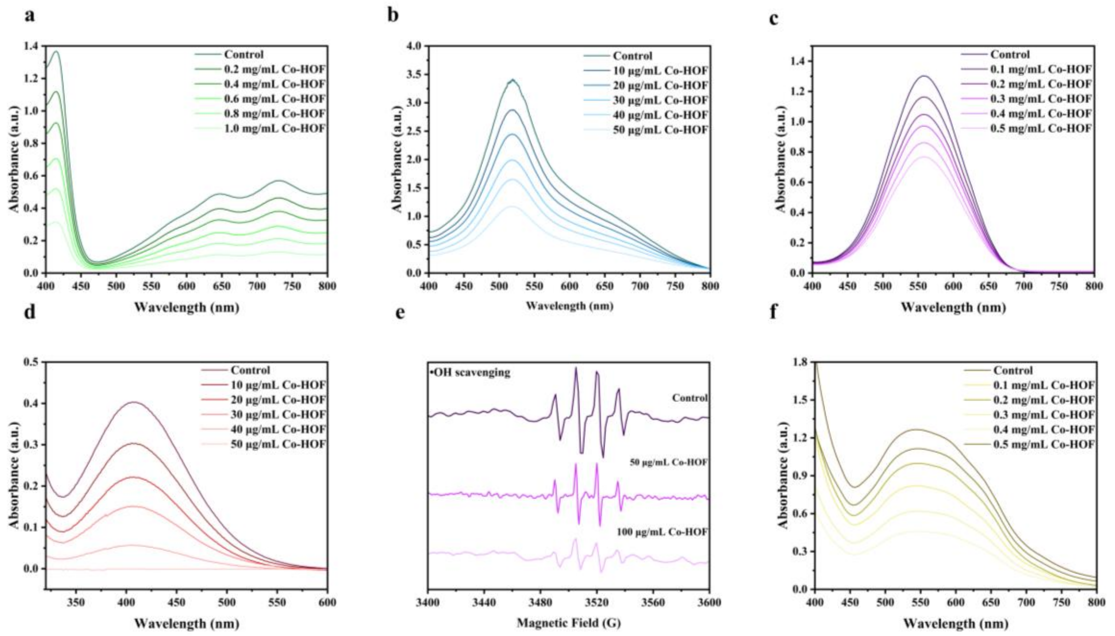

Figure S12. Evaluation of the ROS scavenging capacity of Co-HOF with a) ABTS, b) DPPH, c) PTIO, d) H_2O_2, e) •OH, and f) $O_2^{\bullet-}$ as oxidant, respectively.

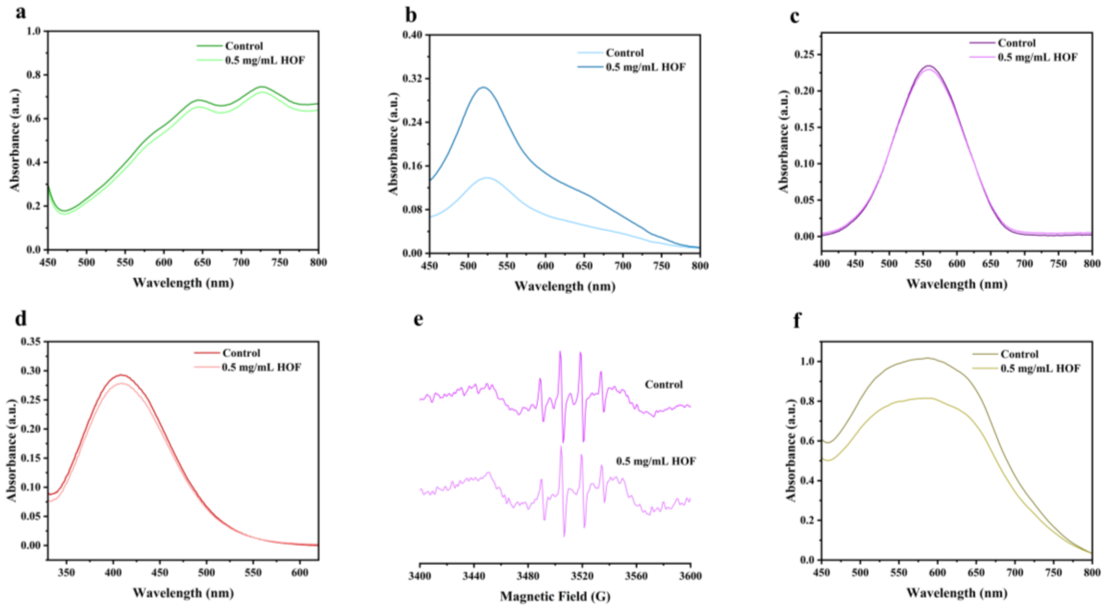

Figure S13. Evaluation of the ROS scavenging capacity of free-base HOF with a) ABTS, b) DPPH, c) PTIO, d) H_2O_2, e) •OH, and f) $O_2^{\bullet-}$ as oxidant, respectively.

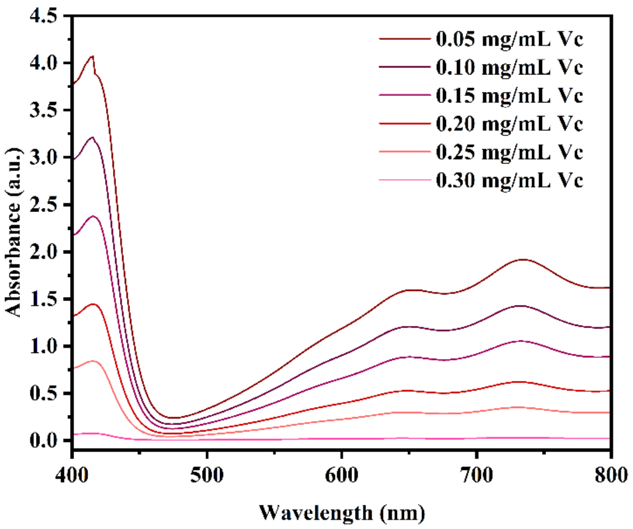

Figure S14. Evaluation of the H_2O_2 scavenging capacity of Vc.

2.11 CV curves measurement

A standard three-electrode cell was used with Ag/AgCl (saturated KCl solution) as reference electrode and Pt electrode as counter electrode. 5 mg sample was dispersed in the solution consisting of 450 μL ethanol and 50 μL 5% Nafion by ultrasonication for 30 min. Then, 100 μL (20 μL each time) of the suspension was pipetted in a 1×1 cm^2 area on an indium tin oxide (ITO) glass and dried naturally at room temperature to serve as the working electrode. All the experiments were conducted in 0.2 M Na_2SO_4 electrolyte at room temperature.

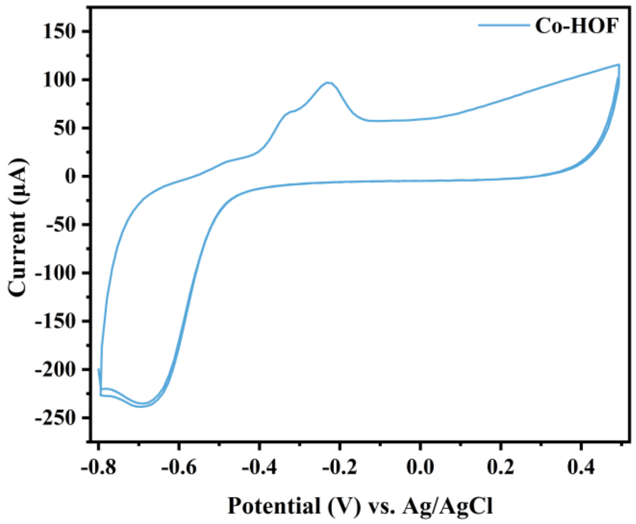

Figure S15. Cyclic voltammetry (CV) curves for Co-HOF, 3-cycle scans at a rate of 50 mV/s.

Standard redox potential[[6]]

Type of redox substances	Standard redox potential (vs. Ag/AgCl)
•OH/H_2O	2.429 V
H_2O_2/H_2O	1.975 V
O_2/H_2O	1.428 V
$O_2^{\bullet -}$/H_2O_2	1.109 V
O_2/H_2O_2	0.894 V
$ABTS^{\bullet +}$/ABTS	0.879 V
DPPH•/DPPH	0.638 V

Table S1. Redox potentials of mentioned reactive oxidative species in this work.

2.12 X-ray photoelectron spectroscopy (XPS) measurements

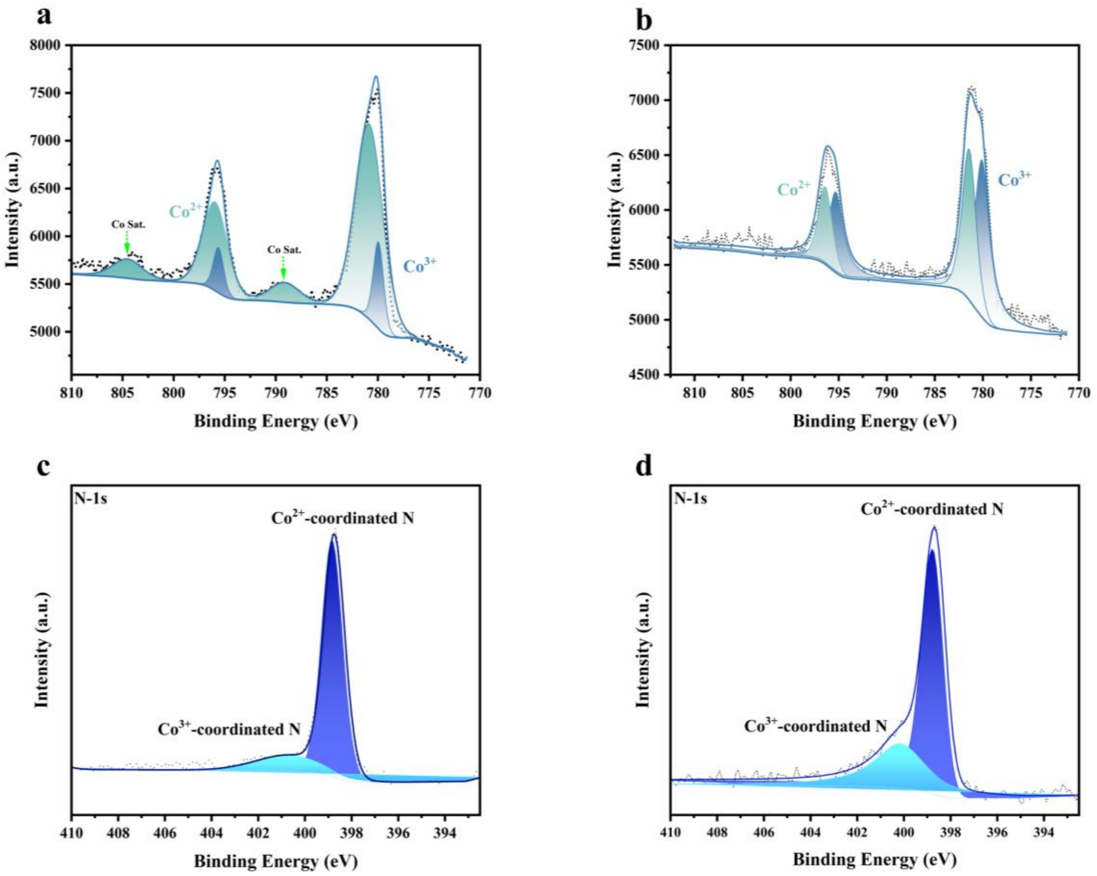

Figure S16. XPS analyses of Co-HOF before and after H_2O_2 scavenging experiments. (**a and c.** before experiments, **b and d.** after experiments)

2.13 Thermogravimetry (TG) curve

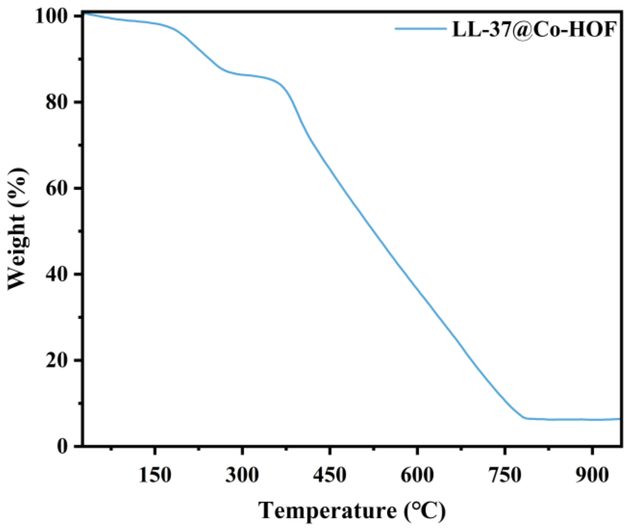

Figure S17. The TG curve of LL-37@Co-HOF.

2.14 Stability test of Co-HOF

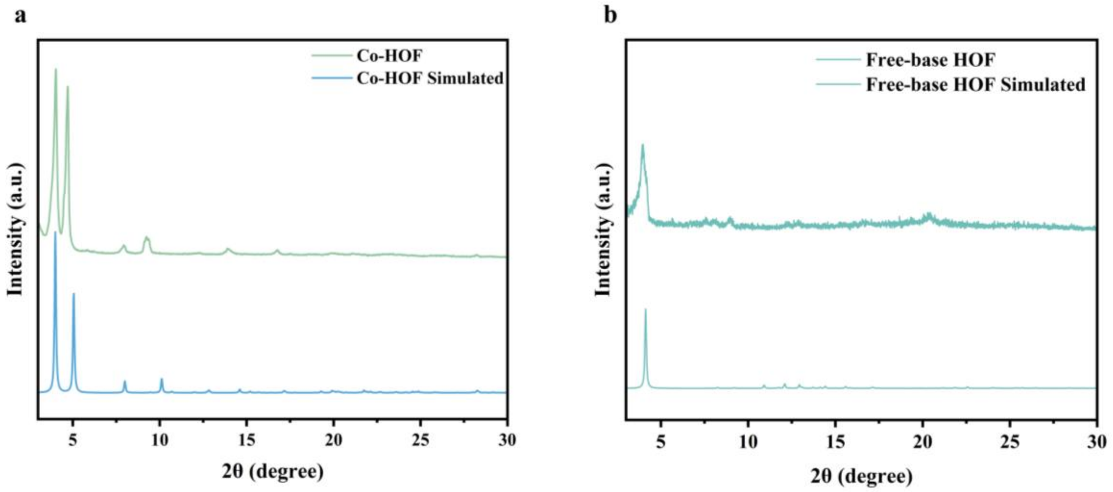

Figure S18. The PXRD pattern of Free-base HOF and Co-HOF.

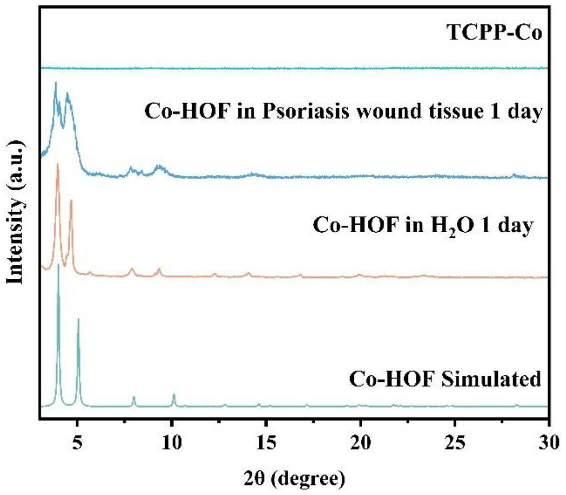

Figure S19. The PXRD pattens of Co-HOF in different conditions.

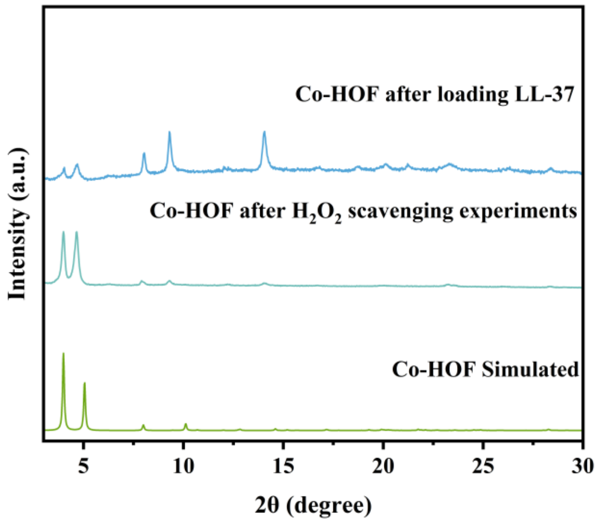

Figure S20. Stability of Co-HOF after being treated with LL-37 and H_2O_2 scavenging experiment.

2.15 HaCaT keratinocytes culture and stimulation

To evaluate the inhibitory properties of Co-HOF, we treated human HaCaT keratinocytes with LL-37/RNA to induce psoriatic responses, followed by Co-HOF treatment. Human HaCaT keratinocytes were purchased from the American Type Culture Collection (ATCC, Cat. # PTA-9170). HaCaT were grown in DMEM/high glucose (Gibco, Cat. # 11960044) containing 10% fetal bovine serum (FBS) in a humidified atmosphere containing 5% CO_2 at 37 °C. Cells at 60%-80% confluence were stimulated with LL-37 (8 μg/mL) and self-RNA (10 μg/mL of RNA extracted from HaCaT cells) for 30 min and then incubated with vehicle (H_2O), Co-HOF (20 μg/mL), free-base HOF (20 μg/mL) and $CoCl_2$ (25 μM), Culture supernatants and cells was collected at 24 hours after with different treatment and subjected to further studies. At least 3 replicates were analyzed for each treatment.

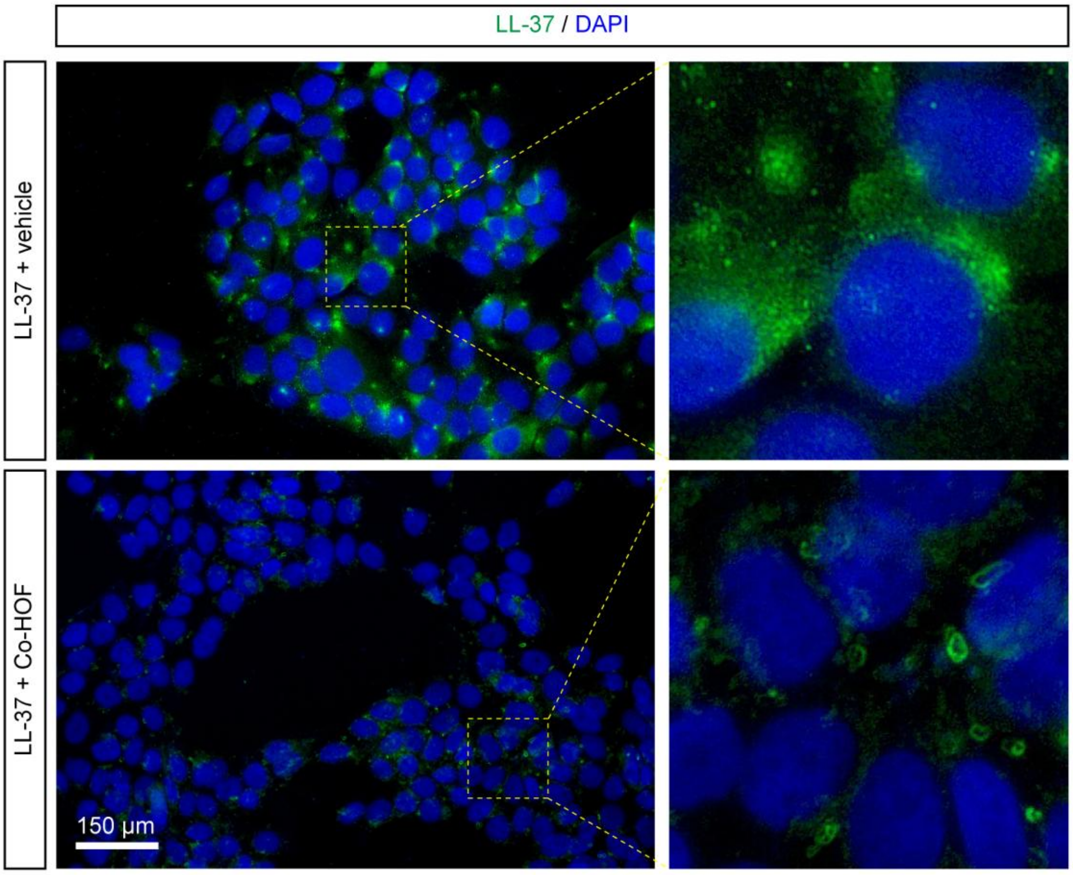

Figure S21. Immunofluorescent analyses of LL-37 in the HaCaT keratinocytes after treatment with LL-37 (8 μg/mL), self-RNA (10 μg/mL) and Co-HOF (20 μg/mL) for 1 h.

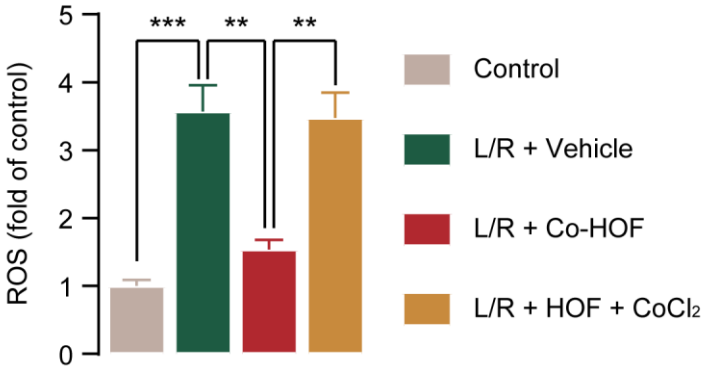

Figure S22. Quantification of ROS in HaCaT cells.

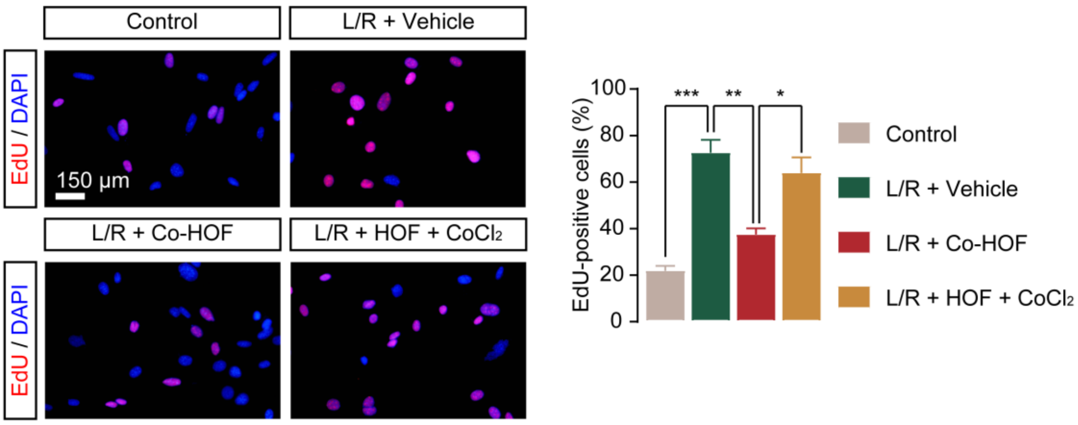

Figure S23. Representative confocal images and quantification of EdU in HaCaT cells.

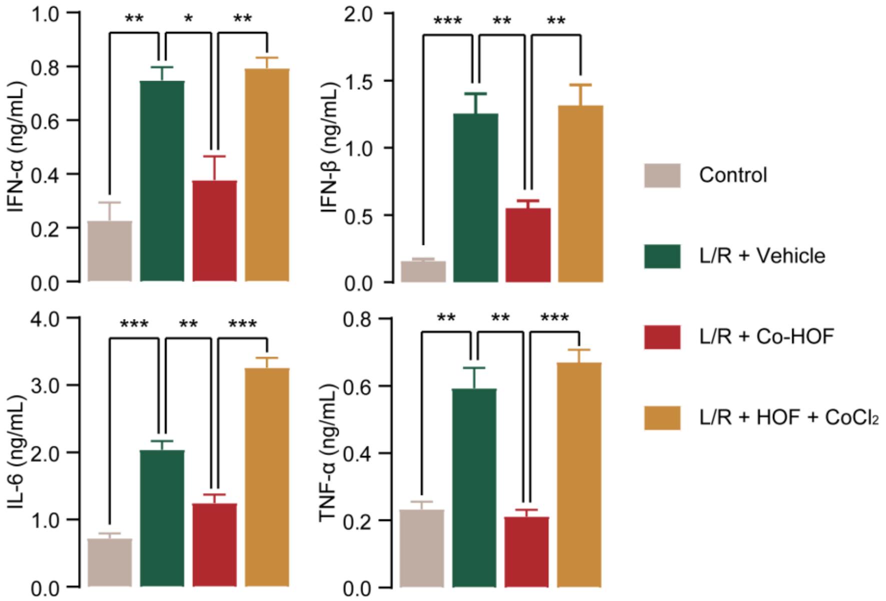

Figure S24. The concentrations of IFN-α, IFN-β, IL-6 and TNF-α in the HaCaT medium were examined by ELISA.

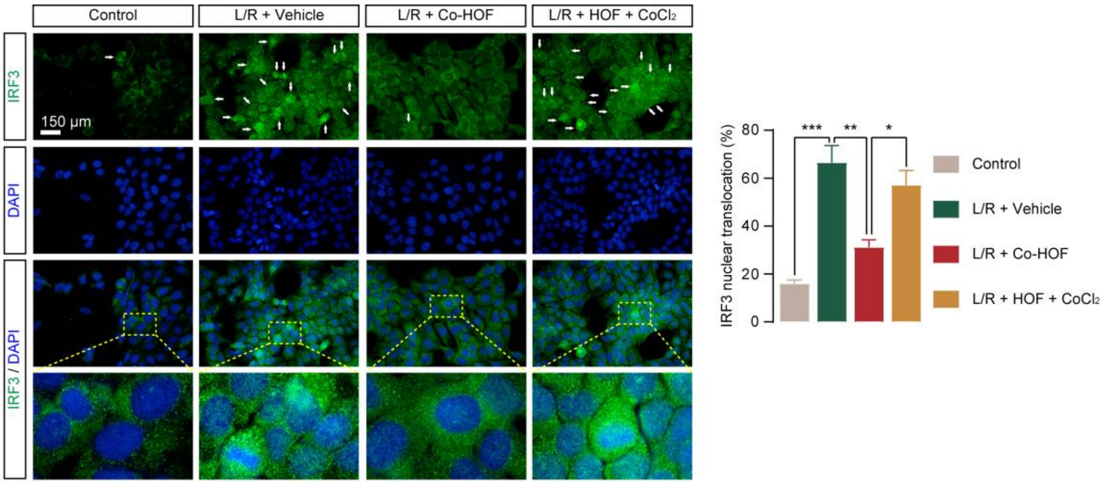

Figure S25. Representative confocal images and quantification of the nuclear translocated IRF3 in HaCaT cells.

2.16 Bone marrow-derived dendritic cells (BMDCs) culture and stimulation

Since LL-37/RNA-mediated dendritic cell (DC) maturation is a key pathogenic factor in psoriasis, we investigated whether Co-HOF could suppress DC maturation and function. BMDCs were cultured using a previously reported method. Bone marrow cells, isolated from both femurs of ICR mice, were cultured in RPMI-1640 (Procell, Cat. # PM150110) with 10% FBS and recombinant mouse granulocyte-macrophage colony-stimulating factor (GM-CSF) (Procell, Cat. # PCK038, 50 ng/mL), and IL-4 (Procell, Cat. # PCK033, 20 ng/mL) for 5 days to generate BMDCs. Immature BMDCs were then incubated with LL-37 (8 μg/mL) and RNA (10 μg/mL) extracted from HaCaT cells for 30 min before treatment with Co-HOF (20 μg/mL), HOF (20 μg/mL), $CoCl_2$ (25 μM) or their water vehicle for 24 hours. Culture supernatants and BMDCs were harvested and subjected to ELISA and flow cytometry studies. At least 3 replicates were analyzed for each treatment.

For fluorescein isothiocyanate-dextran (FITC-dextran) uptake assay, BMDCs were incubated with FITC-dextran (1 mg/mL) at 37 °C for 90 min[7]. Culture supernatants and cells were harvested at 24 h and subjected to further studies. At least 3 replicates were analyzed for each treatment.

In a separate experiment, to generate culture media (CM), BMDCs were treated with LL-37 (8 μg/mL) and RNA (10 μg/mL) extracted from HaCaT cells for 24 hours. After washing with PBS buffers, cells were resuspended in 10% FBS containing DMEM without LL37 and RNA, and cultured for additional 18 hours. Cleared culture supernatant was collected and used as BMDC-CM directly.

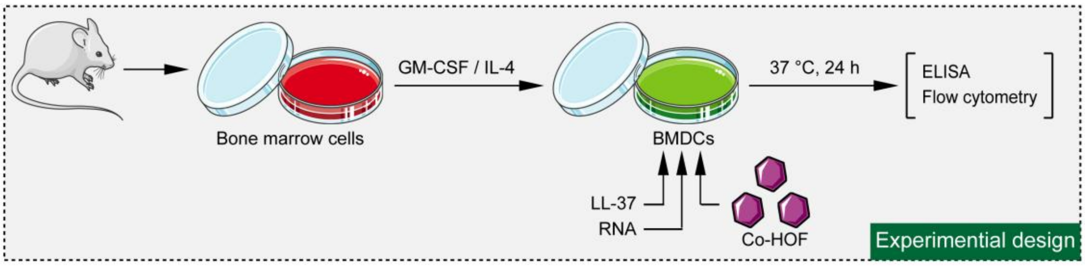

Figure S26. Scheme of treatment with Co-HOF for inhibition of DC maturation.

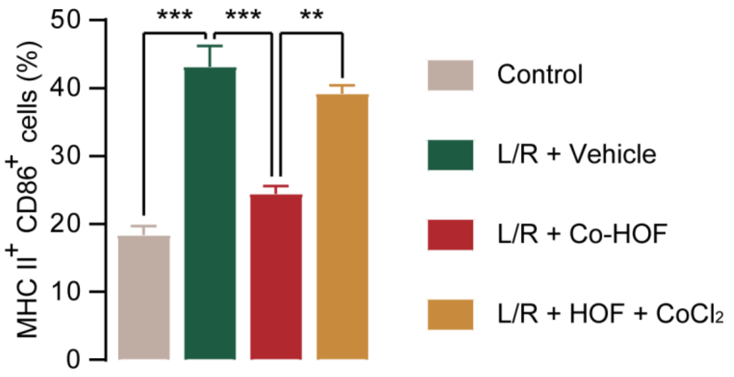

Figure S27. Quantitative analysis of the CD86^{+}/MHC-II^{+} cells in BMDCs

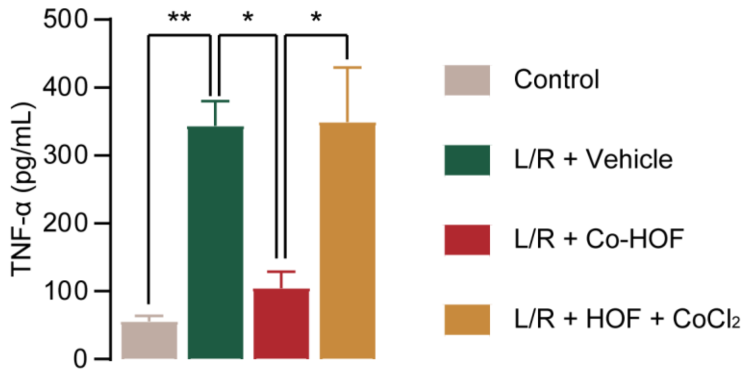

Figure S28. The concentration of TNF-α in the medium was examined by ELISA.

2.17 RAW264.7 macrophage culture and stimulation

Cytokines released by matured DCs can induce inflammation in macrophages. Therefore, we further examined whether Co-HOF could directly inhibit the inflammatory response of RAW264.7 macrophages to cytokines derived from matured DCs. RAW264.7 macrophages were purchased from Procell (Cat. # CL-0190) and were cultured in DMEM supplemented with 10% FBS in a humidified atmosphere containing 5% CO_2 at 37 °C. To stimulate RAW264.7 cells, incubated with CM from BMDCs for 30 min, followed by treatment with Co-HOF (20 μg/mL), HOF (20 μg/mL) and $CoCl_2$ (25 μM), were respectively added to 5 × 10^5 cells in 24 well dishes, for 24 h. Culture supernatants and cells were collected 24 hours after stimulation and subjected to further studies. At least 3 replicates were analyzed for each treatment.

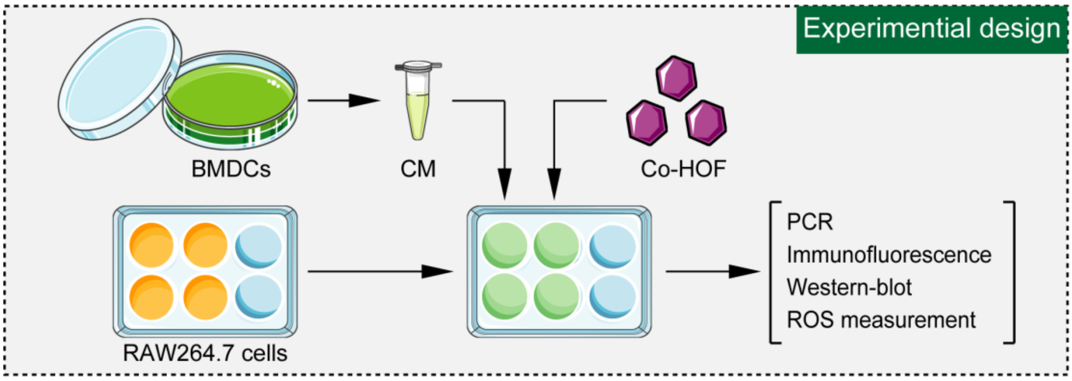

Figure S29. Scheme of treatment with Co-HOF for inhibition of DC-induced macrophages inflammation.

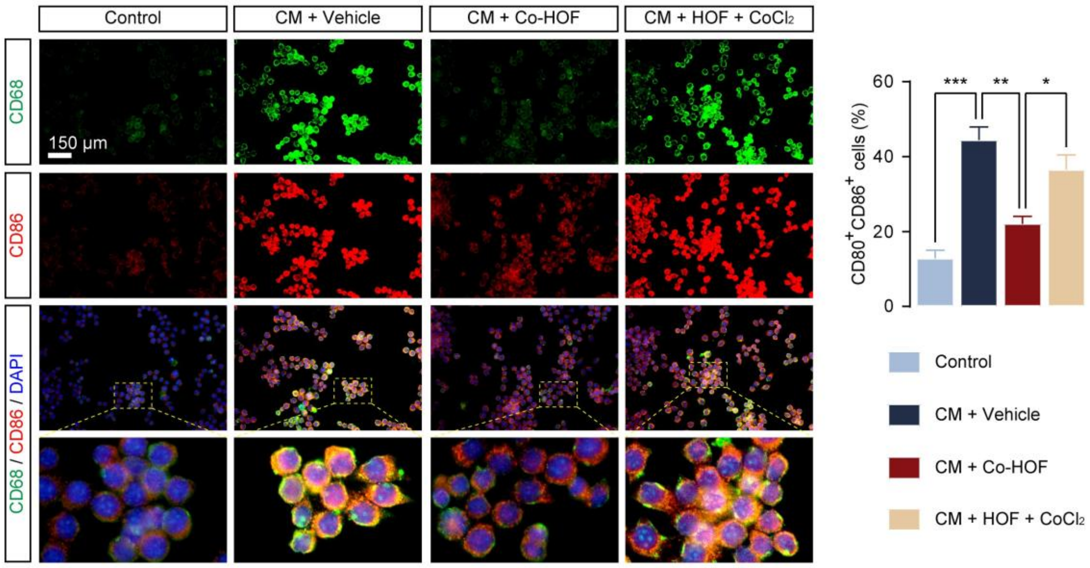

Figure S30. Representative confocal images and quantification of the CD86^{+} and CD68^{+} cells in RAW264.7 cells.

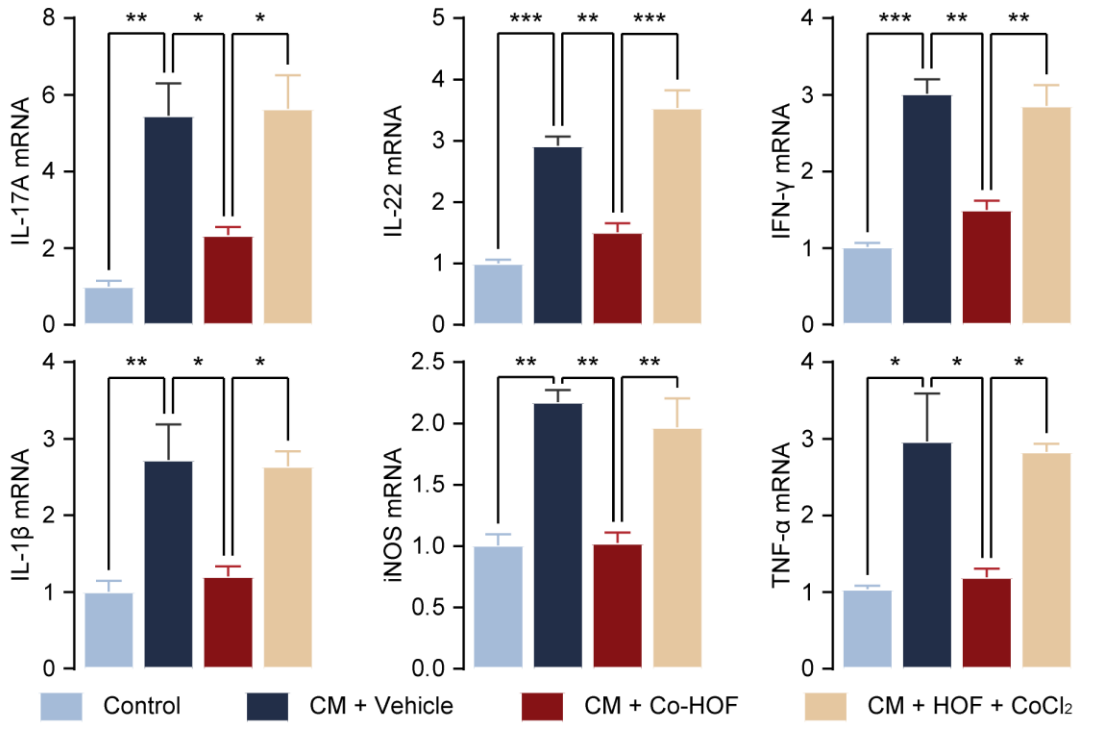

Figure S31. The expression of IL-17A, IL-22, IFN-γ, iNOS, IL-1β and TNF-α in RAW264.7 cells was examined by PCR.

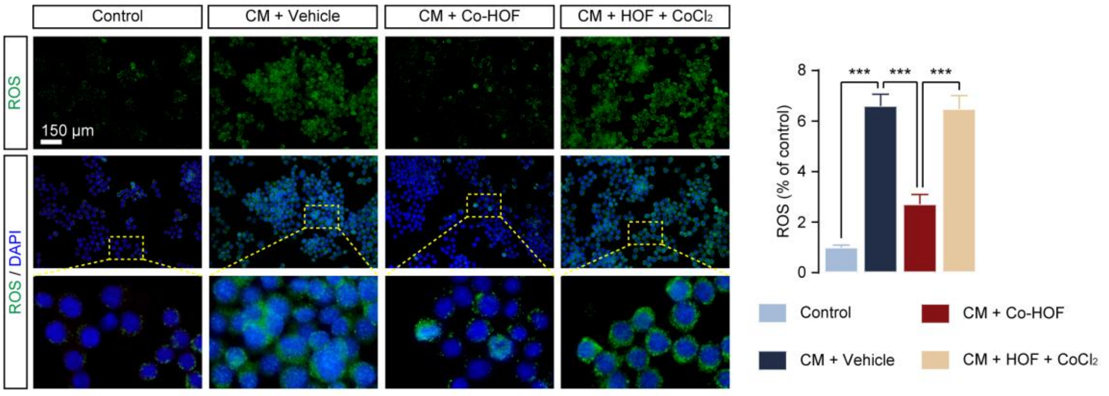

Figure S32. Representative confocal images and quantification of ROS in RAW264.7 cells.

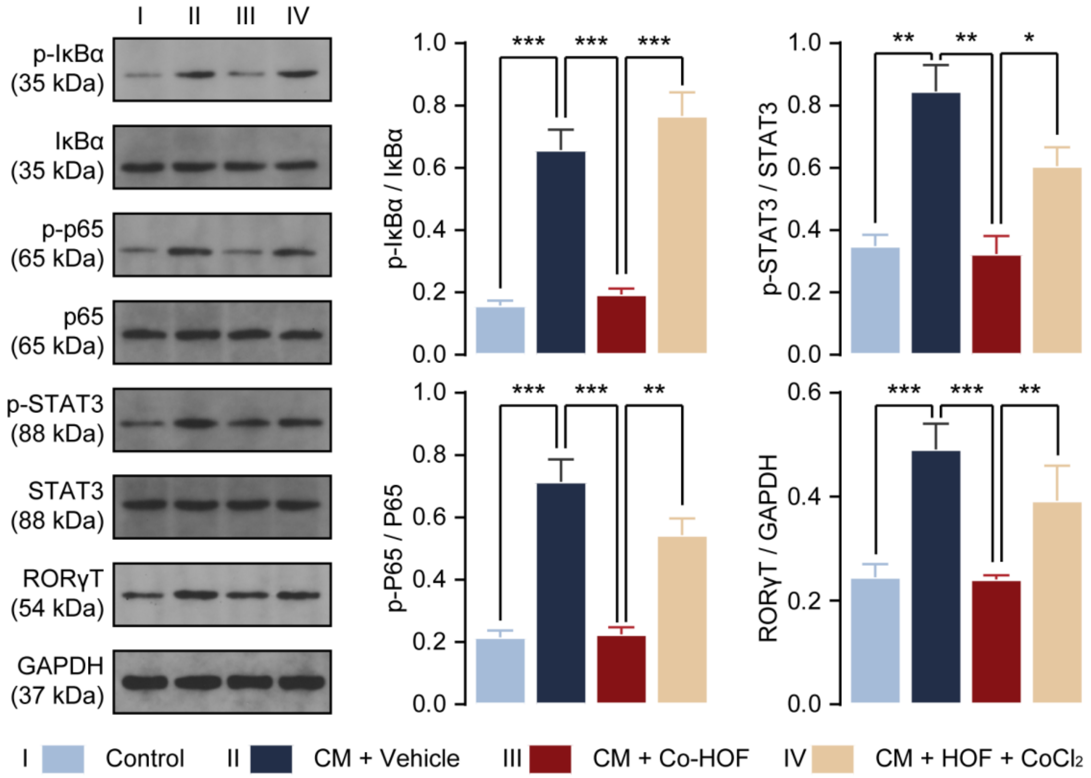

Figure S33. Phosphorylation of p65, IκBα and STAT3 and expression of total p65, IκBα, STAT3 and RORγT in RAW264.7 cells were assessed by western-blotting.

2.18 Cell viability

Cell viability was measured by a cell counting kit-8 (CCK-8) (Dojindo, Cat. # CK04) using a previously reported method[8].

2.19 ROS assay

ROS levels were measured by using 2’,7’-dichlorofluorescin diacetate (DCFH-DA) (Aladdin, Cat. # H131224) or dihydroethidium (DHE) (Aladdin, Cat. # R353922) according to manufacturer’s instructions. Briefly, cells (2 × 104) were incubated with DCFH-DA (10 μM) or DHE (2.5 μM) at 37 ºC for 1 hour and then washed with PBS. Fluorescence was detected using confocal microscopy (Olympus, Japan) and quantified by Image J software.

2.20 EdU assay

EdU assay was performed using BeyoClick™ EdU Cell Proliferation Kit with Alexa Fluor 647 (Beyotime, Cat. # C0075S) following the manufacturer’s instructions. Fluorescence was detected using confocal microscopy and examined by Image J software.

2.21 Western blotting

Western blots were performed using the standard SDS–polyacrylamide gel electrophoresis method. Antibodies against the following proteins were used: anti-LL-37 (Santa Cruz, Cat. # sc-166770, dilution 1:2000); anti-p-TBK1 (Cell Signalling, Cat. 5483S, dilution 1:500); anti-TBK1 (Cell Signalling, Cat. 38066S, dilution 1:1000); anti-p-Akt antibody (Abcam, Cat. ab222489, 1:1000 dilution), anti-Akt antibody (Abcam, Cat. ab8805, 1:1000 dilution); anti-p-IRF3 antibody (Novus, Cat. # NBP3-21582, 1:1000 dilution), anti-IRF3 antibody (Santa Cruz, Cat. # sc-33641, dilution 1:1000), anti-p-p65 antibody (Novus, Cat. # NB100-82088, 1:1000 dilution), anti-p65 antibody (R&D system, Cat. # MAB5078, dilution 1:1000), anti-p-IκBα (Santa Cruz, Cat. # sc-52943, dilution 1:2000); anti-IκBα (Santa Cruz, Cat. sc-373893, dilution 1:500); anti-p-STAT3 (R&D system, Cat. # AF4607, dilution 1:1000); anti-STAT3 (Proteintech, Cat. 10253-2-AP, dilution 1:1000); anti-RORγT (Invitrogen, Cat. # 14-6981-82, dilution 1:1000); anti-GAPDH (Proteintech, Cat. 60004-1, dilution 1:1000).

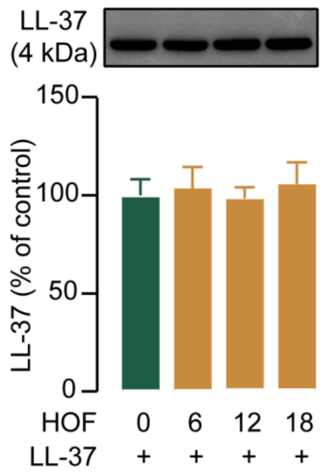

Figure S34. Western-blotting analyses of the content of free LL-37 in the solution (original: 35 μg/mL) after being treated with varying concentrations of free-base HOF (unit: μg/mL).

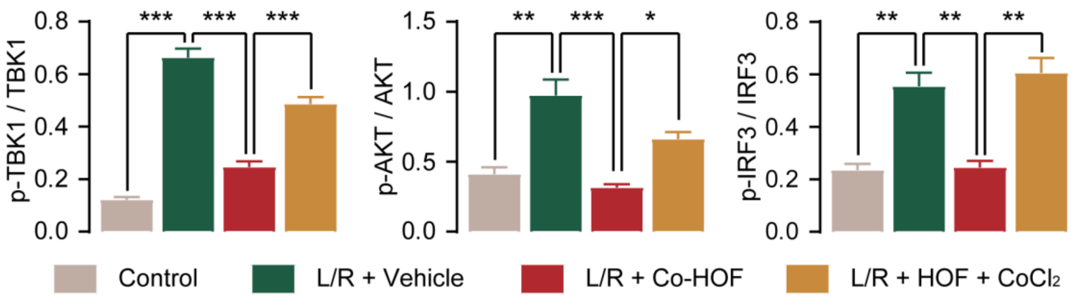

Figure S35. The ratio of p-TBK1 / TBK1, p-AKT / AKT and p-IRF3 / IRF3.

2.22 Reverse transcription-quantitative PCR (RT-qPCR)

Total RNA was isolated and reverse transcribed to cDNA using a previous method[9]. RT-PCR was conducted in a 7300 real-time PCR system (Applied Biosystems), and RNA levels were normalized using GADPH as an internal standard. The primer sequences were used as follows:

IL-17A: ctccagaaggccctcagactac (F); gggtcttcattgcggtgg (R);

IL-22: agcttgaggtgtccaacttc (F); ggtagcactgatccttagcactg (R);

IFN-β: gggactggacaattgcttcaa (F); gcagtacattagccatcagtcacttaa (R);

IFN-γ: ggatgcattcatgagtattgc (F); ccttttccgcttcctgagg (R);

IL-1β: tcgctcagggtcacaagaaa (F); catcagaggcaaggaggaaaac (R);

TNFα: agccccagtctgtatcctt (F), ggtcactgtcccagcatctt (R);

iNOS: cccgtccacagtatgtgaggat (F); cattacctagagccgccagtga (R);

GAPDH: aacagcaactcgcactcttc (F), cctgttgctgtagccgtatt (R);

2.23 Enzyme linked immunosorbent assay (ELISA)

ELISA kits were used to measure IL-6 (Beyotime, Cat. # PI326), TNF-α (Mlbio, Cat. # mIC50536-1), IL-23 (Beyotime, Cat. # PI655), IFN-α (Invitrogen, Cat. # BMS6027) and INF-β ((Beyotime, Cat. # PI568) levels in culture media. The assays were conducted following the manufacturer's instructions.

2.24 Flow cytometry assay

The phenotypes of BMDCs were examined by a BD LSRFortessa X-20 flow cytometer. Single-cell suspensions of BMDCs were incubated for 15 min at 4 °C with PE-conjugated anti-MHC II (Biolegend, Cat. # 116407) and FITC-conjugated anti-CD86 (Biolegend, Cat. # 104705). Hoechst 33342 reagent (BD Biosciences, Cat. # 561908) was used to exclude dead cells. The data were analyzed using FlowJo software.

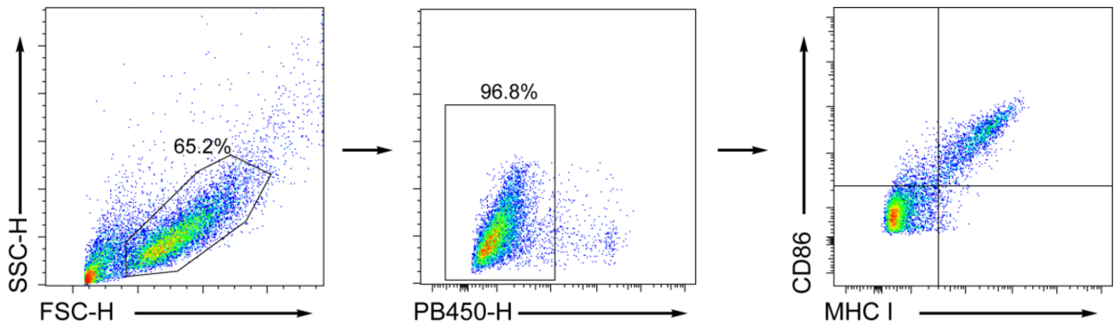

Figure S36. Gating strategies used for flow cytometry.

2.25 Animal

Animal care and experiments were undertaken in compliance with the Guide and Care and Use of Laboratory Animals from the National Institutes of Health (NIH) and ARRIVE, and approved by the Animal Care and Use Committees of Xiamen Institute of rare-earth materials (NO. IREM2024012).

2.26 Imiquimod (IMQ)-induced mouse model of psoriasis

Briefly, male ICR mice (n = 6 per group), 8-10 weeks of age, were anesthetized with 2% isoflurane in 100% oxygen, shaved and treated topically with 63 mg of IMQ cream (3M Pharmaceuticals) once daily for 5 days[10]. Co-HOF (1 mg), free-base HOF (0.74 mg), $CoCl_2$ (0.26 mg) or a comparable water vehicle (0.5 mL) were topically applied 8 h after IMQ treatment once daily for 5 days. Control mice were topically treated with the emollient cream instead of IMQ cream. Mice were sacrificed with isoflurane and tissues were collected at day 5 for analysis.

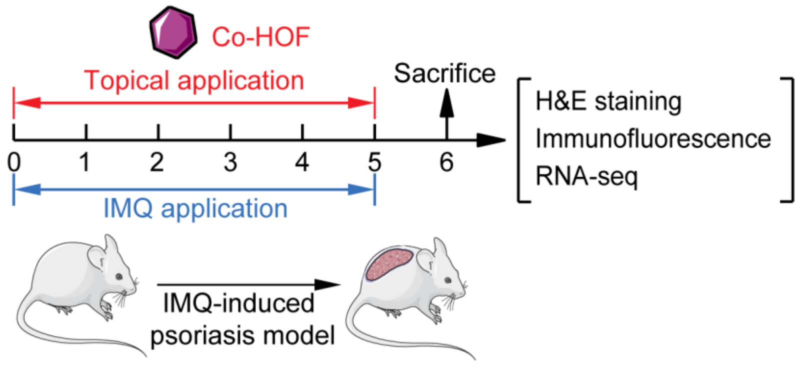

Figure S37. Schematics for the animal experimental procedures.

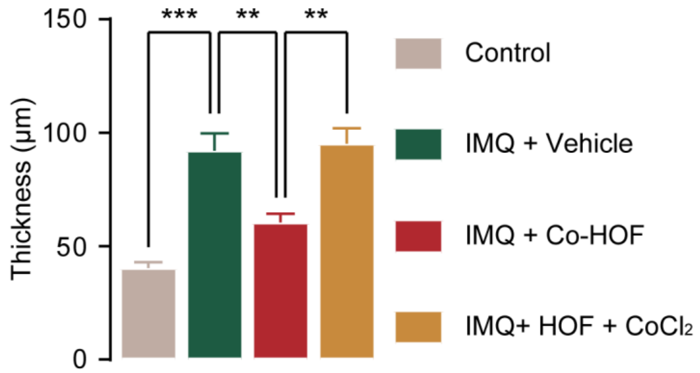

Figure S38. Analysis of the thickness of epidermis in H&E-stained sections.

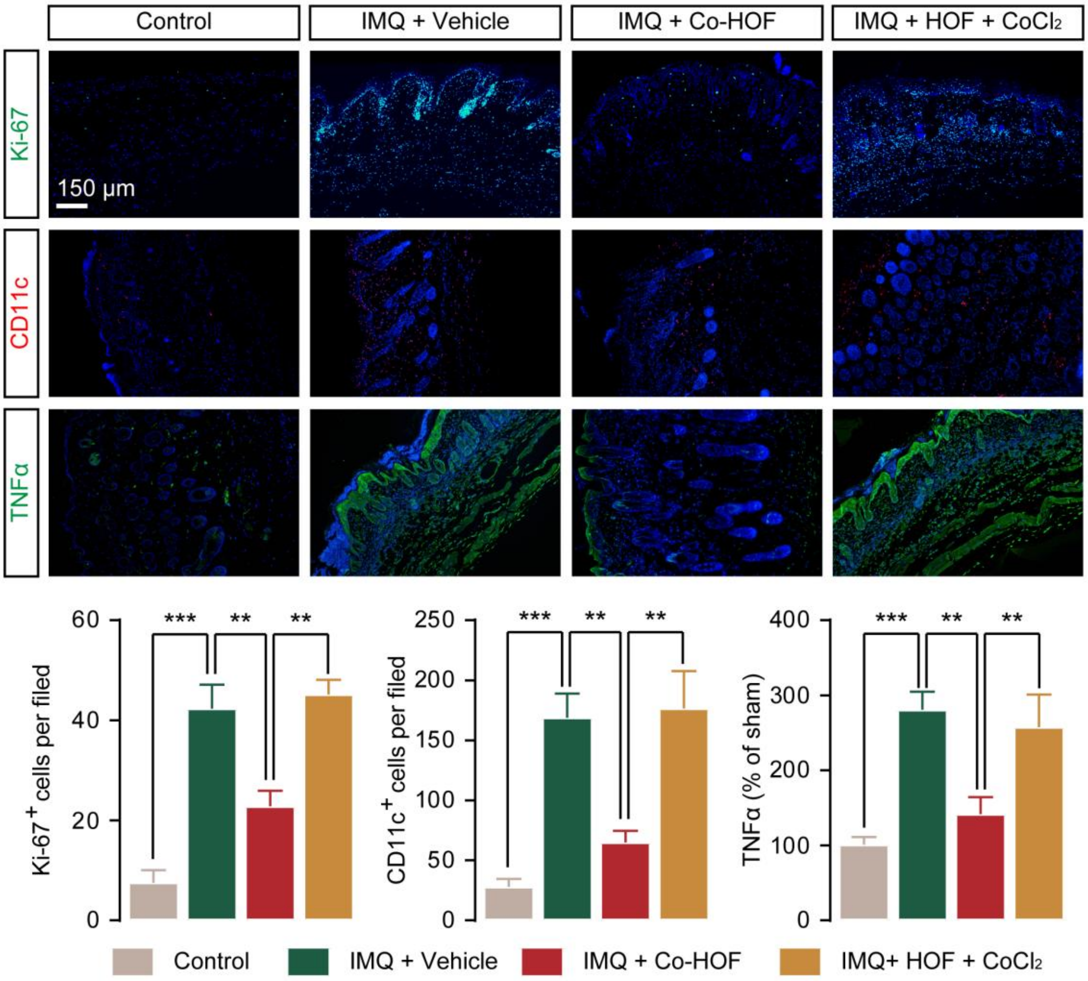

Figure S39. The representative confocal images of Ki-67+ and CD11c+ cells and the expression of TNF-α in the skin area.

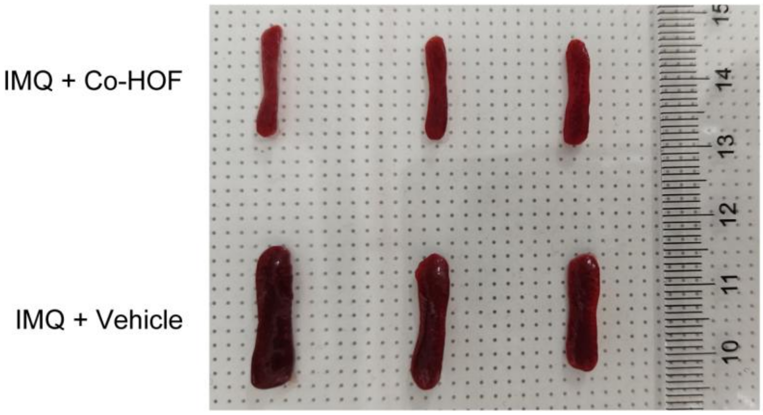

Figure S40. Photograph of the spleen after 6 days' treatment.

2.27 Histology

Specimens from the mouse dorsal skin were fixed with 2% formaldehyde for 24 hours followed by dehydrated and embedded in paraffin. The 5 μm-thick skin slices were obtained using a microtome for consecutive sectioning. Sections were stained with hematoxylin and eosin following standard protocols and scanned using an Olympus microscope. The epithelial thickness was evaluated in more than 3 independent regions.

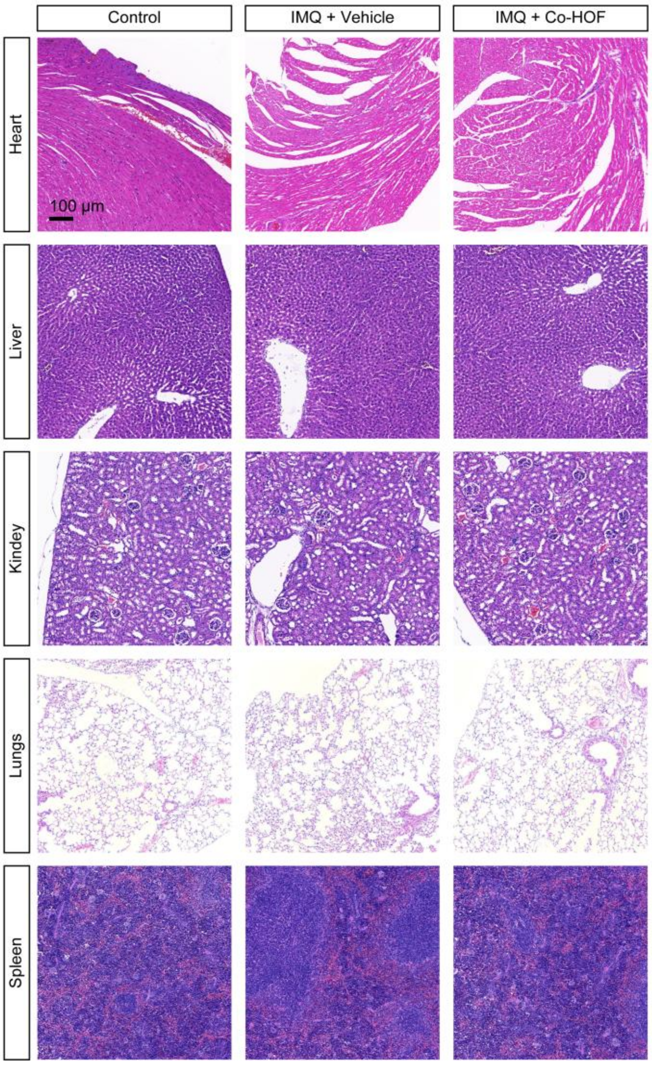

Figure S41. HE staining images of main organs (heart, liver, spleen, lung, and kidney) after 6 days' treatment.

2.28 Immunofluorescence

To further demonstrate the therapeutic effects of Co-HOF on psoriasis, we also study the protein levels of pro-inflammatory factors by Immunofluorescent staining in mice. Paraffin sections of mice as well as HaCaT and RAW264.7 cells cultured on glass slides were examined by immunofluorescent using the standard method. To prevent non–specific binding, slides were blocked with 10% goat serum in PBS for 1 h, followed by incubation overnight at 4 °C with primary antibodies: anti-LL-37 (Santa Cruz, Cat. # sc-166770, dilution 1:500), anti-IRF3 (Santa Cruz, Cat. # sc-33641, dilution 1:1200), anti-CD86 (Proteintech, Cat. # 13395-1-AP, dilution 1:600), anti-CD68 (R&D system, Cat. # MAB10114, dilution 1:1000), anti-Ki-67 (Abcam, Cat. # ab15580, dilution 1:600), anti-CD11c (Cell signaling, Cat. # 93233, dilution 1:300) and anti-TNF-α (Cell signaling, Cat. # 3707, dilution 1:300). After washing with 0.1M PBS, sections were incubated for 1 h at room temperature with goat anti-rabbit IgG-Alexa Fluor 488 (Abcam, Cat. ab150077, dilution 1:800) or IgG-Alexa Fluor 555 (Abcam, Cat. # ab150078, dilution 1:800). Sections were then counterstained with DAPI (Vector Lab, Shanghai, China) for 2 min, mounted and cover slipped with Fluoromount. Fluorescence was detected using confocal microscopy (Olympus, Japan). The numbers of Ki-67^{+} and CD11c^{+} cells were automatically counted using ImageJ software. The mean number of cells was obtained from 3 randomly chosen and nonoverlapping fields (460 × 460 μm) of each section.

2.29 RNA sequencing (RNA-seq) analysis

Total RNA was extracted from the tissue using TRIzol® Reagent according to the manufacturer's instructions (Invitrogen) and genomic DNA was removed using DNase I (TaKara). Then RNA quality was determined by 2100 Bioanalyser (Agilent) and quantified using the ND-2000 (NanoDrop Technologies). RNA purification, reverse transcription, library construction and sequencing were performed at Shanghai Majorbio Bio-pharm Biotechnology Co., Ltd. (Shanghai, China) according to the manufacturer's instructions (Illumina, San Diego, CA). The mice skin RNA-seq transcriptome library was prepared following Illumina® Stranded mRNA Prep, Ligation from Illumina (San Diego, CA) using 1μg of total RNA. The raw paired-end reads were trimmed and underwent quality control by SeqPrep and Sickle using default parameters. The data were analyzed online (www.majorbio.com). All 6 files of transcriptome RNA-seq data (SRA) have been uploaded to NCBI and the BioProject ID is PRJNA1105224.

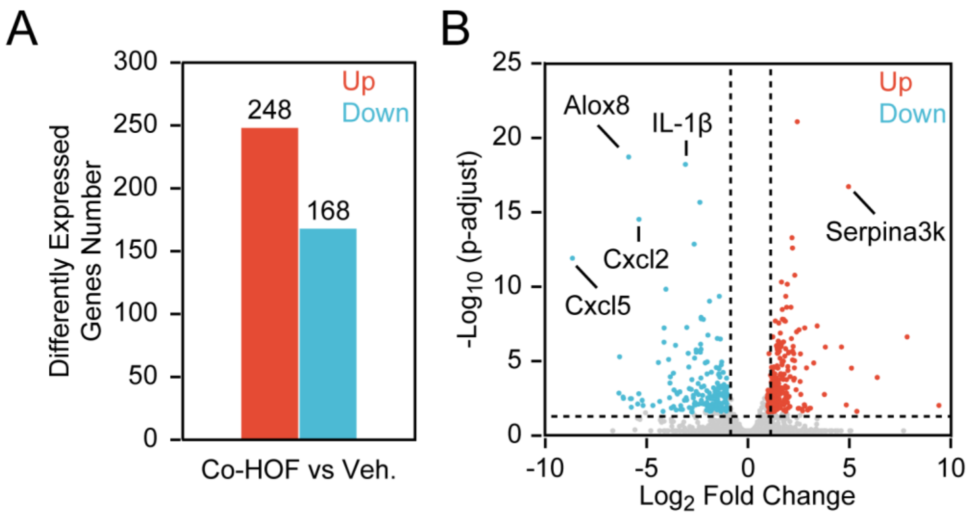

Figure S42. RNA-sequencing of IMQ-induced psoriasis mice skin lesions. (A) Numbers and (B) volcano plots of up-regulated and down-regulated genes of Co-HOF groups, as compared to vehicle groups (n = 3).

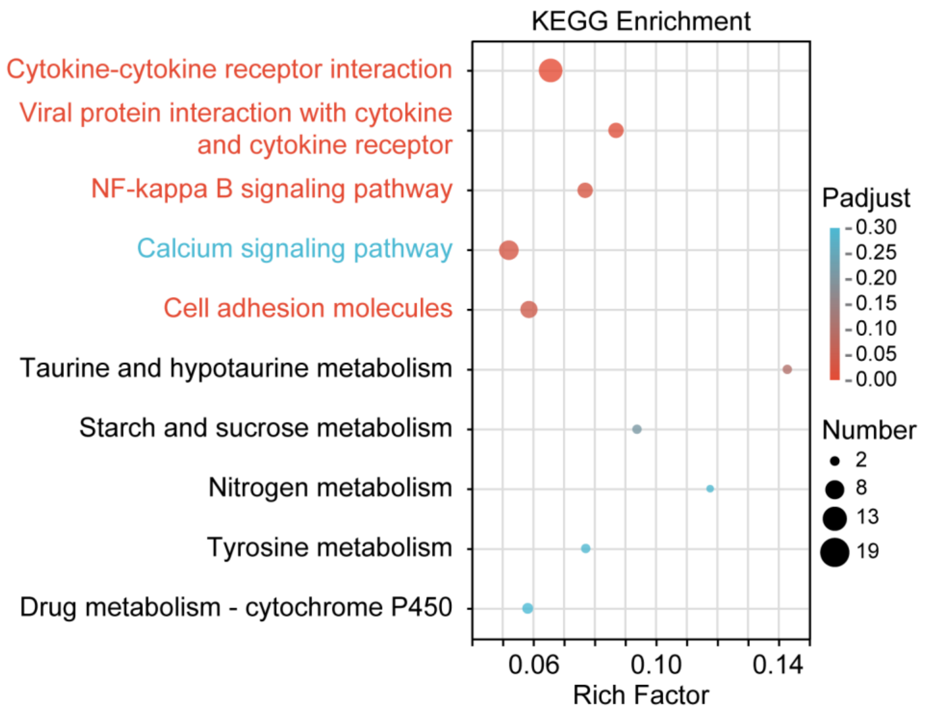

Figure S43. KEGG pathway enrichment analysis (Top 10).

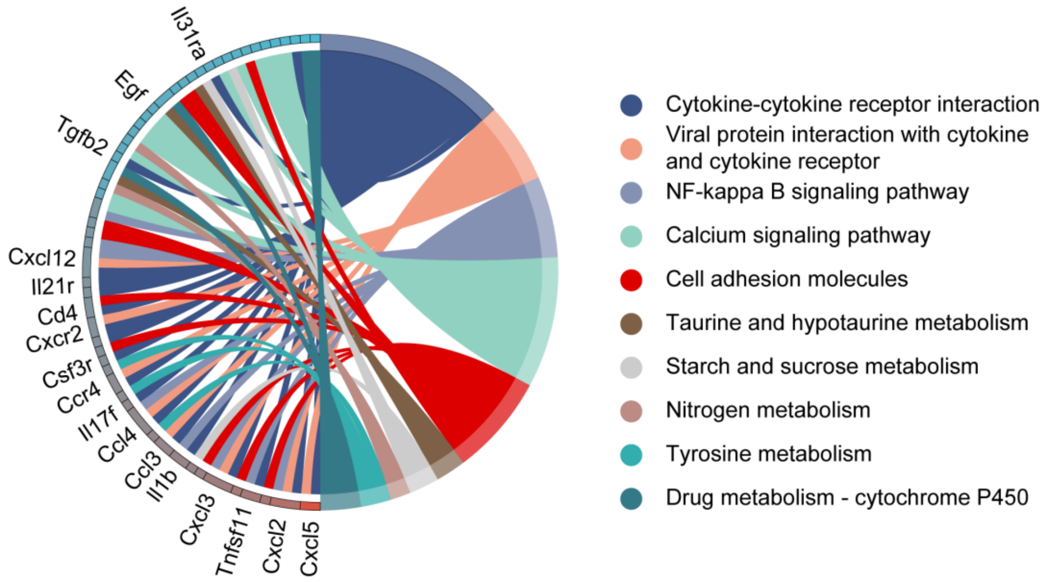

Figure S44. Circular visualization of KEGG enrichment analysis of inflammation related DEGs.

3 Data and statistical analysis

The data were presented as mean ± S.E.M. Statistical analyses were completed using GraphPad Prism version 9.0.0. Two groups were analyzed by the student's t-test (two tailed). $P < 0.05$ was considered statistically significant.

Supplemental References

1. Yin Q, Alexandrov EV, Si DH *et al.* Metallization-Prompted Robust Porphyrin-Based Hydrogen-Bonded Organic Frameworks for Photocatalytic CO_2 Reduction. *Angew. Chem. Int. Ed.* 2021; **61**(6): e202115854.

2. Zhao X, Yin Q, Mao X *et al.* Theory-guided design of hydrogen-bonded cobaltoporphyrin frameworks for highly selective electrochemical H_2O_2 production in acid. *Nat. Commun*. 2022; **13**(1): 2721.

3. Li X, Ouyang X, Cai R *et al.* 3',8''-Dimerization Enhances the Antioxidant Capacity of Flavonoids: Evidence from Acacetin and Isoginkgetin. *Molecules*. 2019; **24**(11).

4. Li XC. Comparative Study of 1,1-Diphenyl-2-picryl-hydrazyl Radical (DPPH•) Scavenging Capacity of the Antioxidant Xanthones Family. *Chemistryselect*. 2018; **3**(46): 13081-13086.

5. Li X. 2-Phenyl-4,4,5,5-tetramethylimidazoline-1-oxyl 3-Oxide PTIO Radical Scavenging: A New and Simple Antioxidant Assay In Vitro. *J. Agric. Food Chem*. 2017; **65**(30): 6288-6297.

6. Liu Q, Ren Y, Jia H *et al.* Vanadium Carbide Nanosheets with Broad-Spectrum Antioxidant Activity for Pulmonary Fibrosis Therapy. *ACS Nano*. 2023; **17**(22): 22527-22538.

7. Wang J, Wang J, Hong W *et al.* Optineurin modulates the maturation of dendritic cells to regulate autoimmunity through JAK2-STAT3 signaling. *Nat. commun*. 2021; **12**(1): 6198.

8. Niu T, Wei Z, Fu J *et al.* Venlafaxine, an anti-depressant drug, induces apoptosis in MV3 human melanoma cells through JNK1/2-Nur77 signaling pathway. *Frontiers in pharm*. 2022; **13**: 1080412.

9. Li Y, Yang L, Chen L *et al.* Design and synthesis of potent N-acylethanolamine-hydrolyzing acid amidase (NAAA) inhibitor as anti-inflammatory compounds. *PloS one*. 2012; **7**(8): e43023.

10. Li Y, Li Y, Xu S *et al.* N-Acylethanolamine acid amidase (NAAA) exacerbates psoriasis inflammation by enhancing dendritic cell (DCs) maturation. *Pharma. Res*. 2022; **185**: 106491.
